# Uric Acid Monohydrate Nanocrystals: An Adaptable Platform for Nitrogen and Salt Management in Reptiles

**DOI:** 10.1021/jacs.5c10139

**Published:** 2025-10-22

**Authors:** Alyssa M. Thornton, Timothy G. Fawcett, Amanda K. Rutledge, Gordon W. Schuett, Jennifer A. Swift

**Affiliations:** † Department of Chemistry, 8368 Georgetown University, Washington, DC 20057, USA; ‡ 265750 International Centre for Diffraction Data, Newtown Square, PA 19073, USA; § 1373 Chiricahua Desert Museum, Rodeo, NM 88056, USA; ∥ Department of Biology and Neuroscience Institute, Georgia State University, Atlanta, GA 30303, USA

## Abstract

Both avian and nonavian reptiles excrete excess nitrogen in solid form—colloquially termed “urates”—as an evolutionary adaptation that aids in water conservation. Yet, there are many open questions regarding the composition, structure, and assembly of these biogenic materials. Here, analyses of urate excretions from ball python (*Python regius*) and 20 other reptile species reveal a clever and highly adaptable system employed to handle both nitrogenous waste and salts. Primitive species excrete urates consisting of 1–10 μm microspheres of turbostratic uric acid monohydrate (UAM) nanocrystals. The nanocrystals’ high surface area and ionizable nature provides a platform to coeliminate substoichiometric concentrations of various salts through surface-ion pairing. In contrast, the granular urates produced by species from more advanced snake lineages are phase mixtures consisting of predominantly ammonium urate hydrate (AUH) and smaller amounts of other crystalline forms. Identification of microspheres as a minor but highly soluble component of these excretions suggests their likely role as reactive precursors to AUH, a hypothesis supported by in vitro experiments. Importantly, this points to a previously unrecognized physiologic function of uric acid, namely the ability to sequester ammonia by transforming it into a solid. The potential implications of this function in other species are discussed.

## Graphical abstract

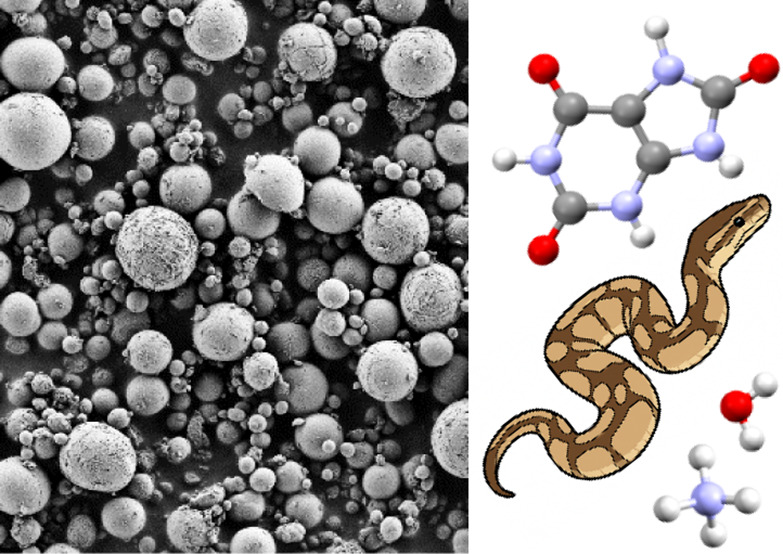

## Introduction

Excess nitrogen resulting from the catabolic breakdown of proteins and purines is usually eliminated as ammonia, urea, or uric acid. Aquatic species typically eliminate ammonia, which is toxic but diluted upon release. Mammals predominantly excrete urea along with substantial quantities of water as urine, but also smaller amounts of ammonia and uric acid that must be carefully regulated. Reptiles and avians are uricotelic, meaning they excrete uric acid in solid form. The solids are colloquially referred to as “urates.” Uricotelism is thought to have conveyed evolutionary advantages, such as enabling water conservation in hot arid climates. It may offer additional reproductive benefits for oviparous species, as uric acid is 10^4^ times less soluble^1,2^ than ammonia or urea, and therefore less likely to adversely affect developing embryos in an enclosed egg environment.^3,4^

In humans, elevated uric acid levels (hyperuricemia)^5^ are more commonly associated with its crystallization *in vivo*, yielding the classic clinical symptom of gout^6–8^ and some types of kidney stones.^9^ While most mammals oxidize uric acid to more soluble compounds for elimination, in humans and other higher primates the uricase enzyme was mutationally silenced approximately 12–14 million years ago.^10–14^ The retention of uric acid provided a presumed fitness advantage,^15^ which has been linked to its potential role(s) as an antioxidant,^16^ in immune signaling,^17^ and in fat storage.^13^ Nevertheless, the dichotomy between the efficient excretion of high uric acid loads in reptiles and avians and the occurrence of crystal-deposition diseases in humans raises interesting questions. (1) How do uricotelic species effectively handle large quantities of this poorly soluble compound? and (2) What is the structure and composition of the “urates” they excrete?

Animal urates have been a subject of scientific inquiry since at least the 19th century,^18,19^ though the first structural analyses of animal urates appeared in the late 1960s. In an examination of bird urates, geologist Folk reported the presence of micron-sized spheres with high aqueous solubility and an X-ray powder diffraction pattern (PXRD) consisting of a single peak.^20^ These observations prompted him to make the erroneous claim that birds do not excrete uric acid, a direct challenge to consensus opinion that elicited a strong response from prominent biologists.^21,22^ Lonsdale and Sutor^23^ followed up with their own studies of a parakeet urate. They confirmed a PXRD pattern with one intense peak at 3.20 Å, but reasoned that the material was a disordered layered form of uric acid dihydrate (UAD). Though a reasonable assumption based on information available at the time, another 26 years would pass before the crystal structure of UAD^24,25^ was determined and found not to have a lamellar structure. In the ensuing years there have been several additional reports of urates from birds,^26–29^ reptiles^26,30–32^ and some insects^31,33–35^ with either a single PXRD peak or a spherical morphology similar to these early reports (Table S1). However, the composition, structure, and properties of these biogenic microspheres have remained open questions.

In our own previous investigations, captive snake species fed identical controlled diets were found to exhibit two general waste elimination behaviors resulting in urates with qualitatively different chemical compositions.^32^ After a meal of rodents, primitive snakes such as boids and pythonids eliminated urates in two intervals—the first as urates-only (U1), and the second as urates and feces in tandem (U2). Both U1 and U2 were voided as a semisolid that dried to a hard pellet with a characteristic powder X-ray diffraction pattern (PXRD) consisting of one intense peak. In contrast, the advanced snake species studied (colubroids) eliminated once after feeding, with urate and feces in tandem. These urates dried to a granular powder with a PXRD pattern consistent with ammonium urates.

Here, based on more extensive studies on urates from ball python (*Python regius*) and a larger group of over 20 primitive and advanced reptile species, we demonstrate that the two urate types—despite their differences—are related through an ingenious and highly adaptable nitrogen and salt management system. Accordingly, all reptiles investigated by us thus far were found to produce microspheres of uric acid monohydrate nanocrystals. Some species eliminate the microspheres directly while others utilize the nanocrystals as reactive precursors to isolate ammonia, as needed, through recrystallization. Recognition of this important function has implications for our understanding of the physiological role of uric acid.

## Results and Discussion

### Urate Microspheres Are Nanocrystalline

The ball python, an ancient snake species,^36^ was selected as a model uricotelic system for its two-step urate elimination process, small size, ease of care, and gentle disposition. On a controlled diet of laboratory mice, adults typically produce U1 within 3–7 days of feeding (Figure 1a), and a smaller mass U2 at 7–15 days after the first elimination. After an especially large meal, one adult male produced a third elimination (U3, urates + feces) on some occasions. SEM images confirmed the urates consist of microspheres ranging in size from 1 to 10 μm in diameter (Figure 1b). U1 and U2 microspheres are indistinguishable in size and physical appearance (Figure S1). Freeze-fracturing revealed that some microspheres are solid while others have a more porous structure with internal concentric rings and/or radial patterns (Figure 1c).

**Figure 1:**
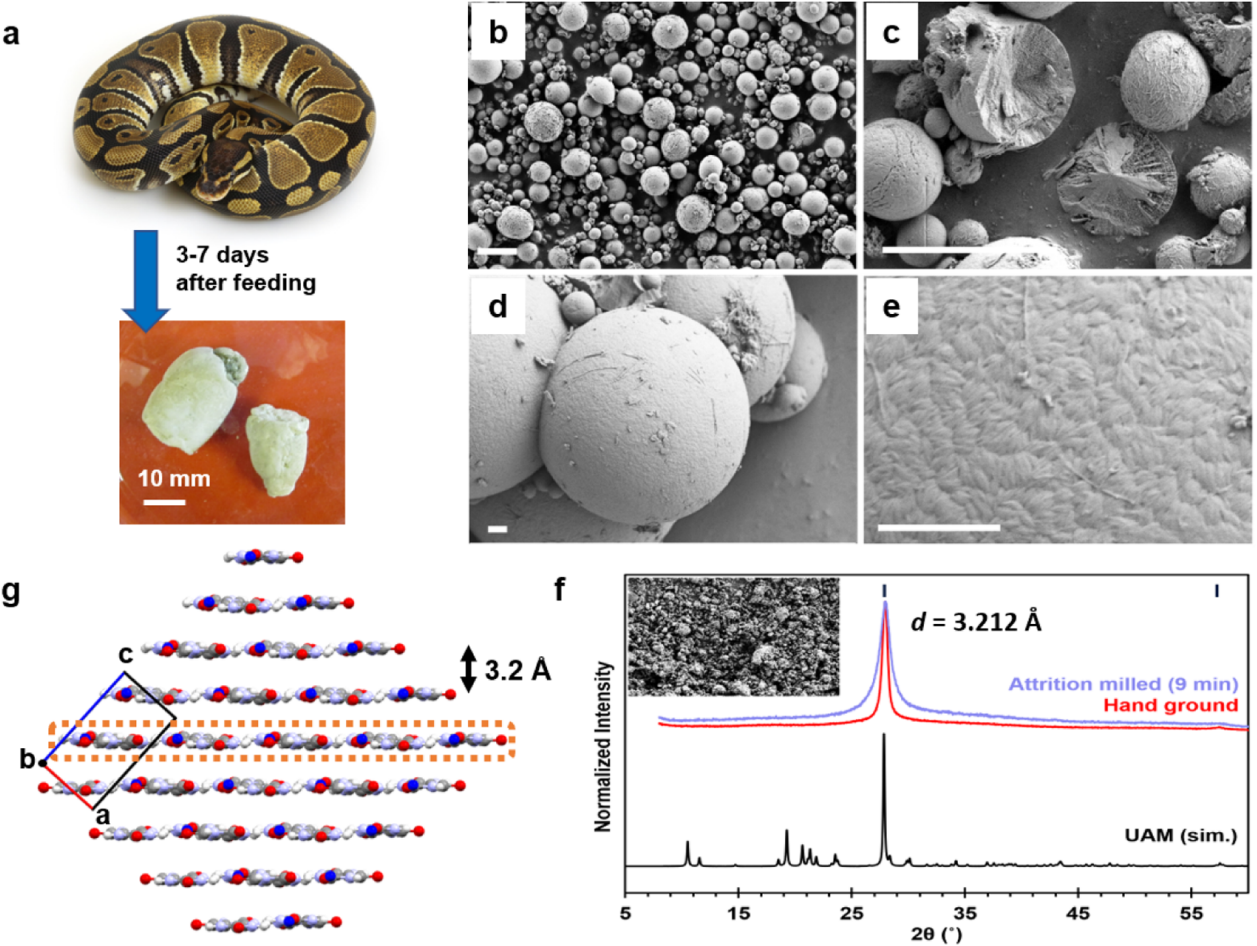
Ball python urates are microspheres of uric acid monohydrate nanocrystals. (a) Ball python (*Python regius*) excretes urates which dry to a hard pellet. (b–e) SEM images of the urates show they consist of 1–10 μm spheres, some of which are covered in thread-like fibers. (c) Freeze-fracturing reveals that some spheres are solid while others have a more open internal microstructure. (d, e) High-resolution SEM images show a granular surface texture with lozenge-shaped particles 40 ± 10 nm in width and 180 ± 60 nm in length. Scale bars (b, c) = 10 μm and (d, e) = 1 μm. (f) The PXRD pattern has one strong peak, a low intensity second harmonic, and very weak diffuse scattering over 2θ = 10–60°. The peak width corresponds to a crystallite size of ∼18–35 nm. Attrition milling fractures the microspheres, and broadens and slightly shifts the peak to 3.212 Å (calibrated against NIST SRM 640c). Data are consistent with a turbostratic layered material. (g) Packing diagram of the UAM structure (refcode: GEJQAO^43^), viewed down the *b*-axis shows the π-stacked (10–2) layers. The water oxygen is colored blue for clarity.

High resolution SEM images of the sphere surfaces showed a texture of densely packed lozenge-shaped particles 40 ± 10 nm in width and 180 ± 60 nm in length (Figure 1e). Analyses of urate microspheres produced by two other ancient snake species, Angolan python (*Python anchietae*) and Madagascan tree boa (*Sanzinia madagascariensis*), had similar textures (Figure S2) confirming that this is a general feature. The uniformity of the nanoparticle sizes points to their likely origination from colloids^37–39^ while the variability in the sphere size suggests a separate mechanism that assembles them into micron-sized packets. We presume that the assembly of nanoparticles into larger spherical units enhances their transport properties, though we do not currently know how or where in the animal this assembly step occurs.

The ball python urates examined had one strong PXRD reflection with a *d*-spacing = 3.191 Å, a low intensity second harmonic, and very weak diffuse scattering over 2θ = 10–60° (Figure 1f). The peak fwhm corresponds to a crystallite size of ∼18–35 nm,^40^ in agreement with the SEM particle sizes. Attrition milling destroyed the microspheres (Figure 1f, inset) but did not generate any additional diffraction lines, thereby ruling out preferred orientation effects in the sample. Milling also slightly shifted the peak position to 3.212 Å and introduced a broadening and asymmetry, both indicative of a 2-dimensional layer structure with turbostratic disorder.^41,42^ Urates from several other species including primitive snakes, lizards and one large avian also produced a similar diffraction pattern with a single intense peak ∼3.2 Å (Figure S3).

Of the many known uric acid and urate salt forms (Table S2), the only one with π-stacked layers matching this *d*-spacing is uric acid monohydrate (UAM)^43^ (Figure 1g). The simulated PXRD pattern of UAM has one intense peak corresponding to the layer separation, with all other peaks having <27% the intensity of the major peak. Turbostratic disorder between the 2-dimensional layers, and perhaps disorder within the layers, would further reduce the intensity of these other peaks. Thermogravimetry, elemental analysis, and FTIR data were also consistent with a monohydrate composition (Figures S4–S6). Ultrahigh performance LC-MS indicated the only measurable organic small molecule in the ball python urates was uric acid (Figures S7 and S8).

Biogenic microspheres of organic crystals (e.g., guanine,^44^ pterin pigments,^45,46^ and isoxanthopterin)^47,48^ have been reported in various animal species, where they play functional roles in optics and/or structural color. In an evolutionary sense, there are potentially interesting connections between microspheres used for optical and excretory functions. However, at present it is not clear how these systems might be related. Unlike biogenic crystals of guanine, which can be a mixture of heterocyclic metabolites,^44^ the absence of small molecules other than uric acid in the excreted urates was notable. There are additional differences in size. Microspheres used for optical applications are, by necessity, fairly consistent. Uric acid spheres identified in the light organ of fireflies^31^ and in chromatophore cells in fish^49^ are fairly uniform in size. In contrast, the excreted urates from ball python and other reptiles consist of microspheres in a range of sizes. The available data on the reptile urates is more consistent with a process wherein UAM nanoparticles of similar size are formed then undergo a second spherification step. This would mean that different uric acid sphere assembly mechanisms may be viable in different contexts.

### Surfaces of Ionizable Nanocrystals May Facilitate Osmoregulation

Reptiles have different mechanisms to eliminate salt, one of which is the excretion of urates and feces.^50,51^ The inorganic content of ball python urates was investigated with semiquantitative SEM-EDX, revealing low levels of K^+^ (<5 wt %) and even smaller amounts of Na^+^, Ca^2+^, and Mg^2+^ (Figure 2). Angolan python and Madagascan tree boa urates had similar K^+^ levels, but no detectable amounts of the divalent ions. Notably, the inorganic ions identified by EDX were present in concentrations well below that expected for stoichiometric salts. Previous EDX studies on urate microspheres from various avian species also showed inorganic ions in varying ratios.^28,52^ Potassium ions were found in the highest abundance in some samples, but in others the levels of Ca^2+^ or Na+ were higher than K^+^. This variation may be due to differences in diet or other metabolic factors.^53^

**Figure 2:**
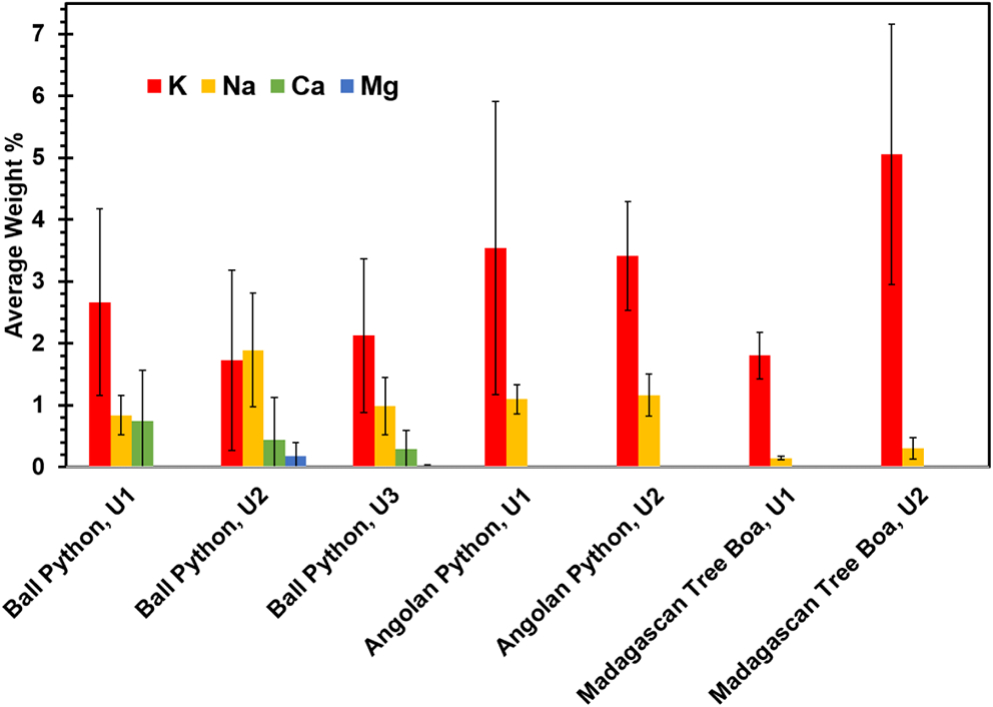
Average ion content of urates produced by primitive snakes: ball python (U1, U2, U3, each *n* = 3), Angolan python (*n* = 2), and Madagascan tree boa (*n* = 2) determined by SEM-EDX.

Nevertheless, if both avian and nonavian reptiles produce nanocrystalline UAM microspheres, their ability to accommodate a range of metal ions in variable concentrations was telling from a functional perspective. Previous studies on anhydrous uric acid (UA) single crystals confirmed that the surfaces bear a slight negative charge owing to the deprotonation of some surface molecules at neutral pH (uric acid p*K*_a_ ∼ 5.4).^54^ Similarly, one expects surface molecules on the UAM nanocrystals to be ionizable. Given the nanocrystals’ high surface area, we infer that a large fraction of alkali and alkaline earth metals in the microspheres are associated with the UAM surfaces. This model does not exclude the possibility that some metal ions reside in other locations, such as in the less ordered spaces between the crystallites or even as minor impurities within UAM. The ability of a high-surface area material to vary its surface charge in response to dietary input would provide a low-energy but highly adaptive platform for osmoregulation. It allows for a range of cation sizes, charges, and concentrations, thereby conveying the ability to eliminate or retain salts, as needed.

### Metastability of UAM Microspheres

It is well-known that uric acid can exist in several different crystalline forms in biogenic environments. For example, while gout deposits are comprised of monosodium urate monohydrate (MSU), in human kidney stones uric acid is most often found as an anhydrate (UA) or dihydrate (UAD). With only one definitive identification of UAM in a kidney stone, this form of uric acid has been considered exceedingly rare.^43^ Ironically, the current study suggests UAM may in fact be the most commonly produced form of uric acid on the planet. So why has UAM not been observed more frequently?

Different crystal forms have different thermodynamic stabilities, introducing the possibility that a metastable phase may transform to lower energy form. For example, synthetic UAD has been shown to recrystallize in solution to UA, a more stable and less soluble form.^55^ To assess the relative stability, the aqueous solubility of ball python UAM microspheres was determined using a fluorescence enzyme assay and directly compared against similar measurements on the forms more commonly observed in calculi (Figure 3a). Replicate measurements of python urates U1, U2, and U3 stored under ambient conditions for ∼4.5 months had equivalent solubilities within experimental error, but were 20–40× more soluble than synthetic MSU and UA. When a second portion of the same U1 was retested after ∼25.5 months, the solubility was roughly half that of the fresher urates due to sample transformation.

**Figure 3:**
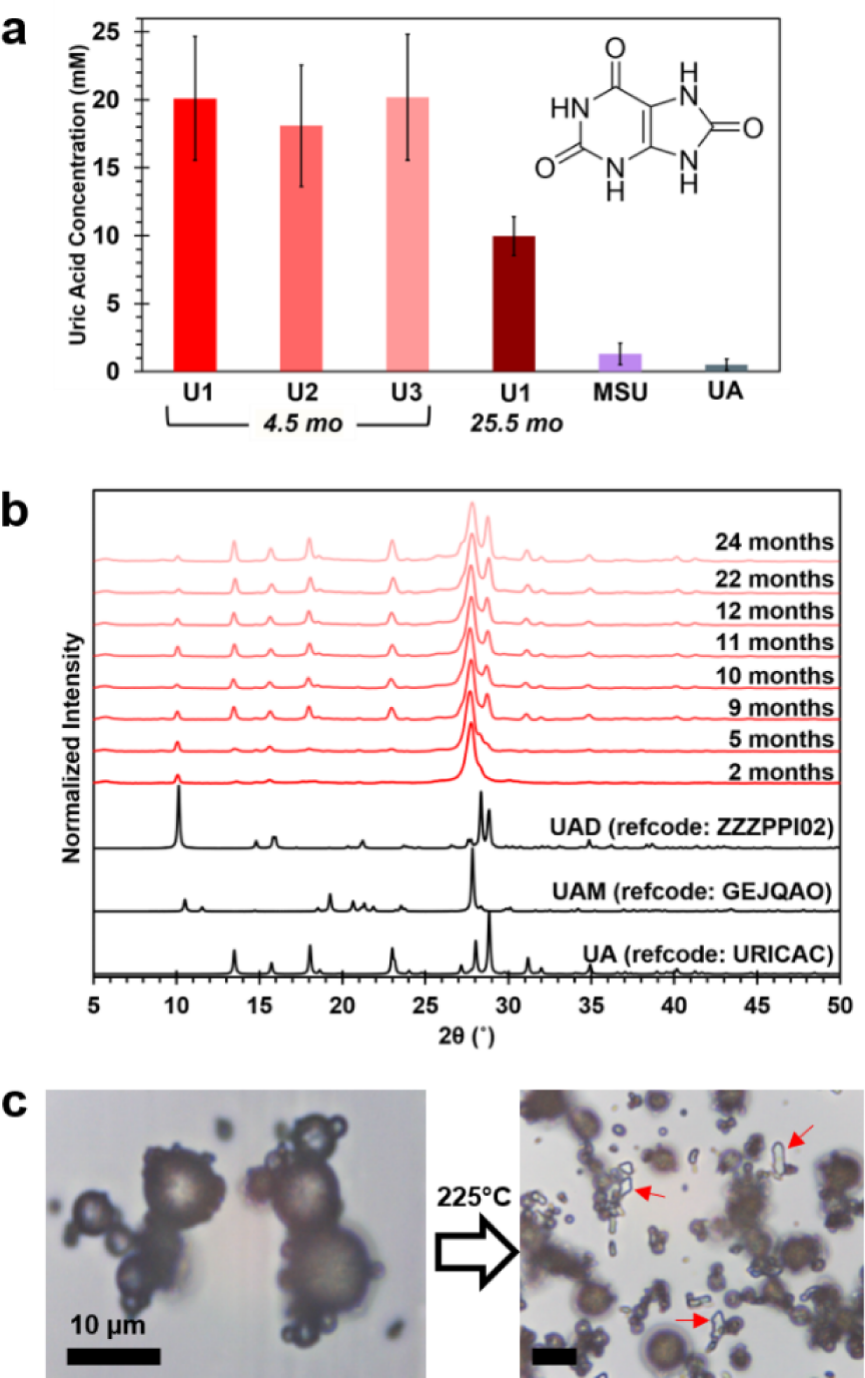
Solubility and stability testing of UAM microspheres. (a) Aqueous solubility of ball python urates, monosodium urate (MSU), and uric acid (UA) measured using a fluorometric enzyme assay. The lower solubility of the sample aged for 25.5 mo is due to its partial transformation to less soluble forms. (b) PXRD patterns of a ball python urate sample aged over an extended two-year period under ambient storage conditions evidence the partial transformation of UAM spheres to more stable UAD and UA forms. (c) Optical micrographs of ball python microspheres before and after heating to 225 °C. Red arrows indicate UA crystals which appear only after heating. Scale bars = 10 μm.

PXRD data collected on the same U1 sample over a 2 year period confirmed that some UAM transforms over time to UAD and UA (Figure 3b). Portions isolated from the larger urate pellet showed that the transformed material was primarily on the exterior while the interior remained as microspheres (Figure S9). Thus, the reduced solubility in aged samples may be attributed to the formation of a less soluble “crust” that essentially encapsulates the microspheres and inhibits their dissolution. However, we note that our analysis of dozens of urates stored long-term under the same ambient conditions indicated that not all samples undergo this transformation at the same rate. Some urates stored for 3 years retained the single peak PXRD pattern characteristic of UAM. On the other hand, small amounts of UAD were sometimes apparent in fresh urates examined <1 week after voiding (Figure S3).

The propensity for the UAM microspheres to transform to UAD and UA is likely related to the original water content of the voided semisolid or the rate at which the pellet hardens. To probe this theory, ball python urates were directly exposed to different environmental conditions. Freshly excreted urates placed in aqueous solution readily recrystallized to UAD and UA.^55^ Heating UAM microspheres in air at 225 °C for several hours also induced a transformation, as evidenced by the appearance of transparent UA crystals among the microspheres (Figure 3c). These accelerated aging conditions both confirm the metastability of UAM and serve to highlight the importance of sample history and environment in structural studies of biogenic uric acid forms.

### Ammonium Urates of Advanced Snakes Are Crystalline Mixtures

Of the >4000 extant snake species,^36,56^ the two-urate elimination pattern is only known thus far in ancient lineages (Boidae, Pythonidae), and far from being ubiquitous. Most modern snake species typically eliminate only once (urates and feces in tandem) and these urate excretions dry to a granular dust (Figure 4a,b).^32^ PXRD analyses of urates from several different colubroid species yielded patterns that seemed a reasonable match to ammonium urate (Figure S10), though biogenic ammonium urates are notorious for their low crystallinity.^57–59^ Recent work identified a second ammonium urate hydrate (AUH) phase, and demonstrated that biogenic ammonium urate is better modeled as a mixture of hydrate and anhydrate forms.^60^ The PXRD patterns of two forms have overlapping peaks at low 2-theta angles, but are distinguished by their most intense peaks at higher angles.

**Figure 4:**
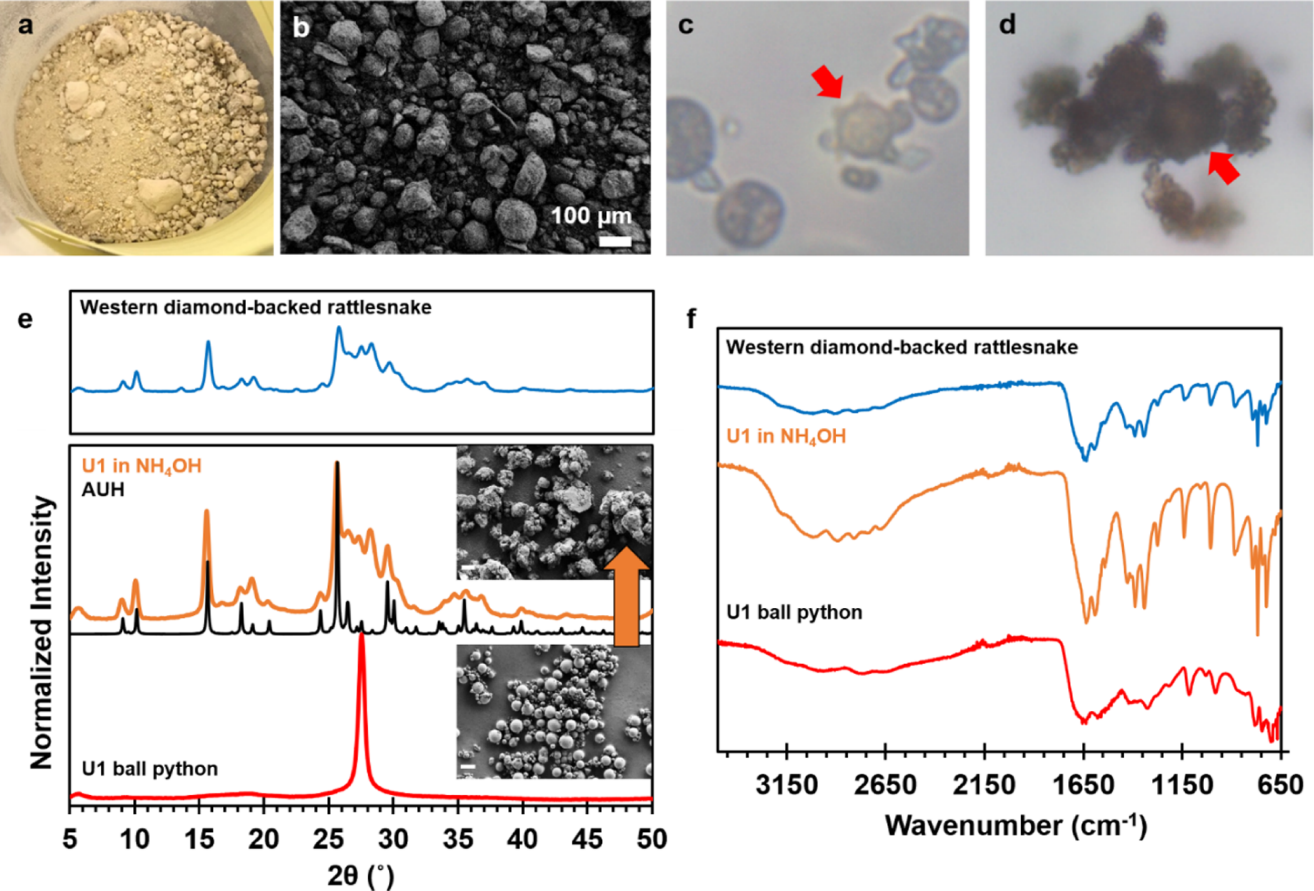
Urates excreted by colubroids dry to a granular dust. Representative excretions from (a) western diamondback rattlesnake (*Crotalus atrox*) and (b) SEM of a urate from Mojave rattlesnake (*Crotalus scutulatus*). Optical micrographs of urates from (c) western diamondback rattlesnake and (d) western copperhead (*Agkistrodon laticinctus*) reveal microspheres (red arrows) as a minor component. Type A microscope oil was used for enhanced contrast at 1000× magnification. (e) PXRD patterns of ball python microspheres before and after immersion in ammonium hydroxide solution (25% in water). Inset SEM images scale bar = 10 μm. (f) FTIR spectra of ball python urate before and after reaction with ammonium hydroxide. Spectra are compared against a urate from a western diamond-backed rattlesnake.

In our microscopic examination of dozens of urates produced by colubroids, we always found smaller amounts of other crystal forms present among the ammonium urate granules. UAD crystals were observed in a few cases, and microspheres were apparent in many others (Figure 4c,d). The identification of microspheres in these samples proved critical to our understanding, as we had previously thought that the different “urate” compositions may derive from different uric acid handling mechanisms.^32^ The presence of UAM microspheres in the ammonium urate “urates” confirmed their commonality across snake species. In fact, the avian literature and our own analysis of a urate from a greater rhea (*Rhea americana*) (Figure S3) point to UAM nanocrystalline spheres as a waste handling system shared across an even wider range of avian and nonavian reptiles. This is presumably a reflection of their common though distant evolutionary ancestry.^61,62^

Yet, these collective observations raise other questions. Of all the possible uric acid forms, why would evolution favor a metastable crystal form as the vehicle for waste management? It may be that the high aqueous solubility is beneficial for water conservation, by enabling greater water reabsorption at a reduced risk of precipitation in vivo. The presence of microspheres in the urates of colubroids pointed to another possible explanation. If UAM nanocrystals were functional precursors to ammonium urate, that would suggest uric acid plays a fundamental role in ammonium management. High solubility would again be advantageous for this function.

### UAM Microspheres Detoxify Ammonia

Animals that excrete ammonium urate must necessarily produce, or have bacteria that produce,^63^ appreciable amounts of ammonia in addition to uric acid. Ammonia is a neurotoxin as well as a potential contact hazard for limbless terrestrial species. Sequestration of ammonia into a solid form would greatly reduce its toxicity. For rattlesnakes and other colubroids in natural settings, the production of granular waste that is easily dispersed in the wind may convey additional benefits such as the ability to avoid predators and ambush prey. Thermodynamics should favor reaction of UAM microspheres with ammonia, since ammonium urate is even less soluble than UA.^64^ The ability to eliminate microspheres directly, or use them as functional precursors to isolate ammonia, as needed, would offer a rather ingenious method to mitigate this hazard.

As proof of concept that UAM microspheres can act as functional precursors to ammonium urate, ball python urates were immersed in a room temperature solution of aqueous ammonium hydroxide. The reacted sample readily took on a granular appearance (Figure 4e) and generated a PXRD pattern strikingly similar to the ammonium urates produced by the colubroids (see also Figure S10). Rietveld refinement of the PXRD data fit to an 86% AUH and 14% microspheres composition, with an *R*_wp_ = 4.79%, good cell precision, and a low residual electron density (Figure S11). The chemical composition was consistent with both FTIR (Figure 4f) and combustion elemental analyses (Figure S12). The thermal properties of reacted spheres were also similar to synthetic ammonium urate hydrate^60^ which is stable below 150 °C but decomposes rapidly when heated above 270 °C (Figures S13 and S14).

### Uricase Silencing—A Hypothesis

This work on reptile urates introduces the possibility that uric acid might play a similar role in ammonia detoxification in other species. While acknowledging that there are significant physiological differences between reptiles and humans, we return to the open question of why humans carry an inactive form of uricase enzyme. If low levels of uric acid protect against rising ammonia levels, that would be a compelling explanation for the fitness benefits associated with uricase silencing.

Both uric acid and ammonia are normal constituents of human bodily fluids. A U-shape association between serum uric acid levels and various diseases has been shown,^65,66^ indicating there are protective benefits at low levels. While UAM is rarely identified in human uroliths,^43^ the hypothesis that uric acid plays a role in detoxifying ammonia does not require UAM per se. The increased solubility of UAM may be significant for reptiles who must also prioritize water conservation in arid climates. For humans, where water is more available, ammonia detoxification would simply require some soluble uric acid. Importantly, even under optimized growth conditions,^67^ synthetic ammonium urate crystals reach maximum sizes <2 μm in diameter. Thus, if such crystals do form in vivo, it is likely they could be eliminated naturally in the urine without adverse effects.

## Conclusions

Our analysis of urates produced by a range of squamate reptiles serves to elucidate key aspects of the very clever adaptable system they employ to manage nitrogenous waste and salt. With dietary controls in place, an appreciation of how environmental storage and aging can affect sample analyses, and the benefits advancements in instrumentation, the current study provides a much more detailed understanding of the structure and function of biogenic urates. How and where the microspheres are assembled remain open and intriguing questions, though the fact that they are produced by a diverse set of uricotelic species suggests a low energy process seemingly optimized by similar selection regimes. The recognition that uric acid plays a role in ammonia management may have broader implications for human health, though clinical studies are needed to fully substantiate the hypothesis.

## Supplementary Material



## Notes

Materials and methods, production and collection of urate samples, urate analysis methods (PXRD, EA, SEM, TGA, DSC, FT-IR, GC-MS), tables of uric acid microspheres in the literature, and crystallographic data for reported uric acid forms (PDF)
