# Supplementary material for: Uric Acid Monohydrate Nanocrystals: An Adaptable Platform for Nitrogen and Salt Management in Reptiles
Source: J Am Chem Soc. 2025 Oct 22;147(44):40236–43. doi: 10.1021/jacs.5c10139 (PMC12593373; doi:10.1021/jacs.5c10139)
Supplement: Supplementary file 1 [file ja5c10139_si_001.pdf]

METHODS AND SUPPLEMENTARY INFORMATION

Uric Acid Monohydrate Nanocrystals: An Adaptable Platform for Nitrogen and Salt Management in Reptiles

Alyssa M. Thornton[1], Timothy G. Fawcett,[2] Amanda K. Rutledge,[1] Gordon W. Schuett,[3,4] and Jennifer A. Swift[1]*

[1] Department of Chemistry, Georgetown University, Washington, DC 20057 USA
[2] International Centre for Diffraction Data, Newtown Square, PA 19073 USA
[3] Chiricahua Desert Museum, Rodeo, NM 88056 USA
[4] Department of Biology and Neuroscience Institute, Georgia State University, Atlanta, GA 30303 USA

Table of Contents

	page
**Materials and Methods**	S4
**Table S1.** Survey of uric acid / urate spheres reported in the literature. (List may not be comprehensive.)	S9
**Figure S1.** SEM images of Ball python urates U1, U2 and U3. There are no obvious visual differences in the sphere sizes or distribution in the U1 and U2 excretions. When U3 excretions are produced after a large meal, some of the microspheres (but not all) exhibit rougher surfaces. The roughening may be related to their partial transformation *in vivo*.	S11
**Figure S2.** (top) High-res SEM images of urates excreted by Ball python, Angolan python and Madagascan tree boa. All scale bars = 1 µm. (bottom) Average grain widths from multiple samples.	S12
**Figure S3.** PXRD patterns of "urates" from 13 ancient reptile and avian species.	S13
**Table S2.** Repeat distances between π-stacked units in uric acid and urate salt forms reported in the Cambridge Structural Database V5.43.	S14
**Figure S4.** Isothermal heating of ball python urates under N_2 at 75°C shows (A) a ~9% weight by TGA. (B) The major peak in the PXRD pattern also becomes slightly asymmetric and shifts to a higher d-spacing = 3.225 Å after heating (calibrated against NIST SRM 640c).	S15
**Figure S5.** Elemental analysis of ball python urates before and after heating at 10 °C /min to 300 °C (red circles) and uric acid (gray circles) compared against the calculated N/C ratios of uric acid anhydrate, monohydrate and dihydrate (black squares). Values are the average of three measurements. The initial sample is assumed to contain some non-crystalline water in addition to structural water.	S16
**Figure S6.** FT-IR spectra of ball python urates (U1, U2 and U3). The FT-IR spectrum of uric acid powder and uric acid dihydrate are included for reference.	S17
**Figure S7.** UPLC chromatograms and mass spectra of water blank and ball python urates sonicated in water in (-) ESI mode. Tandem MS-MS on the small peak at m/z = 357 indicated this was an adduct, $Na^+(urate^-)_2$.	S18
**Figure S8.** UPLC chromatograms and mass spectra of water blank and ball python urates sonicated in water in (+) ESI mode. The small peak at 5.53 minutes has a m/z ratio = 437, though its identity remains unknown.	S19
**Figure S9.** Under normal ambient storage conditions, some ball python urate samples show a slow partial transformation to UAD and UA. (A) From larger aged urate pellets, (B) fractions isolated from the interior retain the single-peak PXRD pattern and	S20

microsphere morphology, while material from the pellet exterior is faceted and has diffraction lines corresponding to more stable uric acid forms.

Figure S10. PXRD patterns of "urates" from snake species that excrete ammonium urate include: Mexican hog-nosed snake (*Heterodon kennerlyi*), Desert kingsnake (*Lampropeltis splendida),* Trans-pecos ratsnake (*Bogertophis subocularis),* Western diamond-backed rattlesnake (*Crotalus atrox)*, Mojave rattlesnake (*Crotalus scutulatus*), Sonoran gopher snake (*Pitaophis catenifer*), Sunbeam snake (*Xenopeltidae*), and western copperhead (*Agkistrodon laticinctus).* Mexican hog-nosed snake, western diamond-backed rattlesnake and Mojave rattlesnake contain small quantities of uric acid dihydrate evidenced by the two sharp diffraction lines at about 2-theta = 28°. S21

Figure S11. Rietveld refinement profiles of ball python spheres reacted in ammonium hydroxide (25% in water). Data was collected at the Advanced Photon Source 17-BM-B and refined to an 86:14 mixture of ammonium urate hydrate:microspheres. The final R_{wp} = 4.79% (P-1: a = 3.661 Å, b = 10.106 Å, c = 10.598 Å, α = 113.91°, β = 90.6°, γ = 92.01°, V = 359.16 Å^3, Z = 2). The difference plot is shown at the top in red. S22

Figure S12. FT-IR spectra of ball python urate before (red) and after (orange) reaction with ammonium hydroxide. Spectra are compared against a urate samples from a western diamond-backed rattlesnake. S22

Figure S13. (top) DSC thermogram of ball python spheres, spheres after reaction in ammonium hydroxide solution, and uric acid. (bottom) TGA of the same ball python spheres and reacted spheres. Both samples were heated at 10 °C/min. S23

Figure S14. Overlay of room temperature synchrotron powder diffraction patterns of reacted microspheres before (black) and after (red) heating to 150 °C. The absence of significant changes in the pattern indicates that the first endotherm in the DSC is due to loss of surface water not structural water. The 2θ scale corresponds to λ = 0.45200 Å. S24

References S24

Materials and methods

Production and collection of urate samples

Postprandial voided uric acid waste samples ("urates") from several taxa of ancient snakes including ball python, (*Python regius*), Angolan python (*Python anchietae),* and Madagascan tree boa (*Sanzinia madagascariensis*), were produced by adult snakes housed at the Chiricahua Desert Museum (CDM). Snakes were offered one to four frozen (thawed) adult laboratory mice (a common strain from a local facility) with water available *ad libitum*. Room temperature was 72–74 °F (~22–23 °C), and cage temperature permitted thermoregulation via a commercial heat strip at one end maintained at 90 °F (32 °C). The Madagascan tree boa (semi-arboreal) had an incandescent basking light (90–95 °F) and was housed in a commercial snake enclosure (61 cm × 61 cm × 61 cm). Ball python and Angolan pythons were housed in enclosures (59 cm × 41 cm × 15 cm). Lighting and supplementary heat were maintained on a 12 h–12 h light-dark cycle. After feeding, the snakes were inspected every 12 h for potential wastes (urates and/or feces). Naturally excreted urates were deposited on clean, fresh, commercial paper toweling. Only the white material that could be separated from the feces (if present) cleanly and reliably were used in subsequent analyses. All urate samples were maintained under ambient temperature and humidity conditions and away from direct light until the time of analysis.

Voided "urates" from other reptile species were provided by Warren Booth, Richard Ihle and Lora Down. These include urates from 2 additional species of pythonids: Macklot's python (*Liasis dunni,* W. Booth) and spotted python (*Antaresia maculosa,* W. Booth); 5 additional species of boid: Argentine boa (*Boa occidentalis,* R. Ihle), Ruschenberg's tree boa (*Corallus ruschenbergerii,* W. Booth), common boa constrictor (*Boa imperator,* W. Booth), Dumeril's boa (*Acrantophis dumerili,* R. Ihle), and emerald tree boa (*Corallus caninus,* W. Booth), and 2 lizard species: glass lizard (*Ophisaurus attenuatus,* L. Down) and tegu lizard (*Tupinambus* sp., R. Ihle). Tara Harris and Drew Foster from the Phoenix Zoo collected samples from greater rhea (*Rhea americana*). The animal diet and storage conditions of these samples prior to our acquisition and testing are unknown.

Optical microscopy

Optical micrographs were collected using a compound trinocular microscope (Swift SW380T) with a total magnification range between 40X and 1000X. Some high magnification images (600X

and 1000X) were taken under Type A microscope oil. This helped to disperse samples as well as change the refractive index to allow for clearer particle imaging. A microscope slide with 600-, 150- and 70-micron calibration dots was used to validate the magnification power.

Scanning electron microscopy (SEM)

Samples were mounted on 12 mm conductive carbon tape attached to aluminum SEM mounting stubs (M. E. Taylor Engineering, Inc.). A Zeiss Supra 55-VP scanning electron microscope (Zeiss, Germany) was operated under vacuum at 1 kV accelerating voltage using secondary electron mode detection. To minimize charging at higher magnification, some samples were coated with a 50 Å gold layer by high vacuum magnetron sputtering (CVC Products).

Ball python urates were freeze-fractured by placing hand-ground samples between two SEM stubs on carbon tape. The stubs were held together and immersed in liquid nitrogen for 30 seconds. The frozen spheres were fractured by separating the stubs before imaging. Energy dispersive X-ray (SEM-EDX) data from all spheres were collected using 20 kV. X-ray spectra were collected using a capture time of 90 seconds per point, and dead time was less than 15%. Data was averaged from at least 5 points on separate spheres.

Uric acid solubility assay

The aqueous solubility of snake urates, uric acid, and monosodium urate (MSU) were measured using a commercial uric acid fluorescence assay kit (Cayman Chemical Company, Ann Arbor, MI). Anhydrous uric acid (UA) was used as received (Aldrich, 99+%). Monosodium urate monohydrate (MSU) was prepared according to published methods[68] by combining 170.3 mg uric acid, 818.3 mg NaCl, and 1.250 mL 1 M NaOH in 100 mL 18 MΩ water, heating to dissolve, and crystallization at 37 °C for 1 day. Phase purity was confirmed by PXRD.

A standard calibration curve was generated from eight solutions with known uric acid concentrations ranging from 0 (control) to 10 µM. UA, MSU and biogenic assay samples were stirred in 18 MΩ water for 2 h, then centrifuged, filtered, and diluted with assay buffer. Each well of a 96-well plate was filled with 105 µL diluted assay buffer, 15 µL fluorometric detector, 15 µL sample and 15 µL enzyme mixture. After a 15-minute incubation at room temperature, the fluorescence was measured with an Infinite 200 PRO plate reader (Tecan) using an excitation wavelength of 530 nm and an emission wavelength of 590 nm.

Replicate measurements for each sample were averaged and the standard error of the mean determined. The control sample value was subtracted from all other values to obtain the corrected fluorescence (CF). Calculations of uric acid concentration in each sample were determined as described in the assay:

$$\text{Uric acid } (\mu\text{M}) = \left[\frac{\text{CF-(y-int)}}{\text{slope}}\right] \times \text{Well plate dilution} \times \text{Sample dilution}$$

The concentration was calculated using dilution factors 50-500. All error was calculated using propagation of error.

Powder x-ray diffraction (PXRD)

PXRD data were collected on ground urate samples using a Bruker Apex DUO X-ray diffractometer (Cu *K*α radiation, transmission mode, 50 kV, 30 mA current, 3-60°, Kapton capillary) or a Bruker D2 Phaser 2nd generation X-ray diffractometer (Cu *K*α radiation, reflection mode, 300 W, 0.02° step, 0.5 s steps, rotate at 15 rpm, 8-70°, zero background Si disk). In some cases, and internal NIST-calibration standard (SRM 640c Si powder) was added to the sample for peak position calibration.

Variable temperature synchrotron powder X-ray (sPXRD) data were collected at the Advanced Photon Source (APS) beamline 17-BM-B with a λ = 0.45200 Å (27.4302 keV). Manually ground samples were loaded in Kapton capillaries (Cole-Palmer 95820-07, OD: 1.0 mm, ID: 0.9 mm), stoppered with quartz fiber filters, placed in a flow cell,[69] and rocked 15° (at ~2-3° per minute) while under a constant He gas flow (5 mL/min). With a beam size = 300 µm, detector distance = 600 mm, and 1 second per image exposure time (summed over 10 images), sPXRD patterns were collected every ~30 seconds while heating at 10 °C/min. GSAS-II[70] was used for image processing and integration.

PXRD patterns were compared against all known crystal forms of uric acid (anhydrate,[71] dihydrate,[25] and monohydrate[43]) and urate salts available in the Cambridge Structural Database (CSD)[72] as well as experimental patterns in the 2023 releases of the ICDD's PDF-4+ database[73] and the PDF-4/Organics database.[74]

Thermal analysis (DSC, TGA)

Differential scanning calorimetry (DSC) data were collected on a TA Instruments Discovery DSC 25 using 3 – 5 mg sample in capped but unsealed aluminum pans. Samples were heated at 10 °C/min from room temperature to 400 °C.

Thermogravimetric analysis (TGA) was obtained on a TA Instruments SDT Q600 using a nitrogen flow rate of 50 mL/min. All experiments used 3.0-5.0 mg of ground sample in open ceramic pans heated isothermally at 75 °C or ramped at 10 °C/min.

Elemental analysis (EA)

Elemental analysis was performed using a Perkin Elmer 2400 CHN Series II Elemental Analyzer with acetanilide as a calibration standard. All samples were run in triplicate.

Infrared spectroscopy

Fourier-transformed infrared (FT-IR) spectra of samples hand-ground in a mortar and pestle were recorded on a Perkin Elmer Spectrum-Two spectrophotometer equipped with a UATR-TWO diamond ATR attachment. Scans were collected on each sample over a 600–4000 cm^{-1} range, with each spectrum representing an average of ten scans.

Stability testing of ball python urates

The stability or transformation of ball python urates as a function of environmental parameters was assessed as a function of time (under ambient conditions), temperature, and exposure to water and ammonia.

Extended ageing: PXRD data was collected on the same ball python urate sample on a near-monthly basis for a period of 24 months.

Heat: Ball python "urates" were heated for different periods of times and temperature in either a drying oven or under N_2 in a TGA. After heat treatment, samples were analyzed with optical microscopy, PXRD, TGA and/or EA.

Milling: Ball python urates were attrition milled with 64 agate grinding elements for period up to 9 minutes, then analyzed with PXRD and SEM.

Water: Fresh ball python urates were immersed in water, and subsequently analyzed with optical microscopy and PXRD.

Ammonia: Ball python urates were immersed in a 25% ammonium hydroxide solution (Acros Organics) and held at room temperature for 15-24 h. Samples were vacuum filtered and ground in a mortar and pestle before analysis by PXRD, sPXRD, SEM, TGA and/or EA.

Ultra-high performance liquid chromatography with quadrupole time-of-flight mass spectrometry (UPLC-MS)

Ball python "urates" were sonicated in DI water, and 20 μL of the solution was added to 1 mL 50/50 water/acetonitrile. The solution was vortexed and injected into an ACQUITY UPLC BEH C18 column (130 A, 1.7 μm, 2.1 mm x 50 mm) in a water/acetonitrile gradient (+ 0.1% formic acid) mobile phase at 0.5 mL/min. The column eluent was directly introduced to the G2 mass spectrometer for quadrupole-time-of-flight MS in negative (capillary voltage = 2.00 kV, sampling cone voltage = 30 V) and positive (capillary voltage = 3.00 kV, sampling cone voltage = 30 V) electrospray ionization. The desolvation gas flow was 1000 L/hr and desolvation temperature was 500 °C. The cone gas flow was 25 L/hr and the source temperature was 120 °C. The data was acquired in the sensitive MS mode with 0.300 sec sans and an interscan time of 0.014 sec. Accurate mass was maintained by infusing Leucine Enkephalin (556.2771 m/z) in 50% aqueous acetonitrile (2.0 ng/mL) at 20 μL/min via the Lockspray interface every 10 sec. Data was acquired in centroid mode with 50-1200 m/z mass range for TOF MS scanning.

Rietveld refinement

Multi-phase Rietveld refinement methods were performed in the whole pattern fitting (WPF) module in JADE Pro using general procedures described in reference 60. The unit cell, scale factors and an overall temperature factor were initially refined. Peak profiles were refined in later cycles, and atomic parameters and site occupancies were selectively refined in the final steps. A refineable 5th order polynomial was applied to eliminate the Kapton contribution.

Our structure model for ammonium urate hydrate (AUH) was based on an initial energy minimized structure calculated using VASP[75] and DFT. Multi-phase refinement of ball python urate reacted in ammonium hydroxide yielded a 86:14 ratio of AUH : microspheres, and a final R_{wp} = 4.79 %.

Table S1. Survey of uric acid / urate spheres of specific avians, reptiles and insects reported in the literature. List may not be comprehensive.

**Group**		**Sphere size (µm)**	**PXRD (Å)**	**Ref.**
Aves	Chicken, goose, turkey, pigeon, sea gull, sparrow, starling, parakeet (in USA)	2-8	3.20-3.23	20
Aves	Pigeons, sparrows, magpies, galahs, parakeets, white and black cockatoos, ravens, eagles, emus, seagulls (in Australia)	2-8	3.20-3.23	20
Aves	Parakeet (budgerigar)		3.20 + weak 1.6	23
Aves	Greater road runner	2-10		26
Aves	Red-tail hawk	1-10		26
Aves	Gambel's quail	2-8		26
Aves	White leghorn (domestic)	2-12		26
Aves (likely)	White material from dust traps collected over 8 yrs	2-10	3.2	27
Aves	Domestic fowl (*Gallus gallus)*	5-12		[52]
Aves	Domestic fowl (*Gallus gallus)*	0.5-10		28
Aves	Northern cardinal (*Cardinalis cardinalis*)	0.5-10		28
Aves	Rufous-sided towhee (*Pipilo erythrophthalmus*)	0.5-10		28
Aves	Chipping sparrow (*Spizella passerine*)	0.5-10		28
Aves	House sparrow (*Passer domesticus*)	0.5-10		28
Aves	Gray catbird (*Dumtella carolinensis*)	0.5-10		28
Aves	Wood thrush (*Hylocichla mustelina*)	0.5-5		28
Aves	Barn swallow (*Hirundo rustica*)	0.5-10		28
Aves	Ovenbird (*Seirus aurocapillus*)	0.5-10		28
Aves	Chicken (*Gallus Gallus*)		3.28	29
Aves	Helmeted Guineafowl (*Numida Meleagris*)		3.28	29
Aves	Mixed parrot species		3.28	29
Aves	Blue and Gold Macaw (*Ara ararauna*)		3.28	29
Aves	Greater Rhea *(Rhea americana)*		3.24	This work
Snake	Garter snake (*Thamnophis elegans vagrans*)	2-10		30
Snake	*Boiga dendrophila, Constrictor constrictor, Crotalus viridis helleri, Eryx johni, Heterodon platyrhinos, Naja naja, Pituophis melanoleucus catanifer*	Ureteral urine all spheres, cloacal urine		30

		spheres + other shapes		
Snake	King snake	1-10		26
Snake	Madagascan tree boa (*Sanzinia madagascariensis*)		3.22	32
Snake	Angolan python (*Python anchietae*)		3.23	32
Snake	Ball python (*Python regius*)		3.21	32
Snake	Dumeril's boa *(Acrantophis dumerili)*		3.25	This work
Snake	Argentine boa *(Boa occidentalis)*		3.25	This work
Snake	Emerald tree boa *(Corallus caninus)*		3.25	This work
Snake	Ruschenberg's tree boa *(Corallus ruschenbergerii)*		3.25	This work
Snake	Common boa *(Boa imperator)*		3.24	This work
Snake	Macklot's python *(Liasis mackloti)*		3.24	This work
Snake	Spotted python *(Antaresia maculosa)*		3.25	This work
Lizard	Desert iguana (*Dipsosaurus dorsalis*)	2-10		30
Lizard	*Cnemidophorus tigris, Diposaurus dorsalis, Eublepharis maculatus, Heloderma horridum, Sauromalus obesus, Uma notata, Varanus salvator, V. varius*	Ureteral urine all spheres, cloacal urine spheres + other shapes		30
Lizard	Horned lizard	1-30		26
Lizard	Blue tongue skink	1-13		26
Lizard	Bearded dragon	2-10		26
Lizard	Gecko (*Hemidactylus stejnegeri*)		3.22	31
Lizard	Glass lizard *(Ophisaurus attenuatus)*		3.24	This work
Lizard	Tegu lizard *(Tupinambus* sp.*)*		3.24	This work
Sauria	Spiny lizard	3-20		26
Chelonia	Tortoise (*Testudo graeca*)	2-7		26
Insecta	Firefly light organ (*L. cerata*)	0.7(regular) but some up to 4	3.20	31
Insecta	Firefly light organ (*Diaphanes citrinus*)		3.19	31
Insecta	Silkworm larvae (*Bombyx mori*)		3.23	31
Insecta	Armyworm larvae (*Pseudaletia separata*)			35
Insecta	Silkmoth (*Hyalophora cecropia*)			33
Insecta	Cockroach (*Periplaneta americana*)	1-40		34
Insecta	Cockroach (*Blatta orientalis*)	1-40		34
Insecta	Cockroach (*Nyctibora lutzi*)	1-40		34

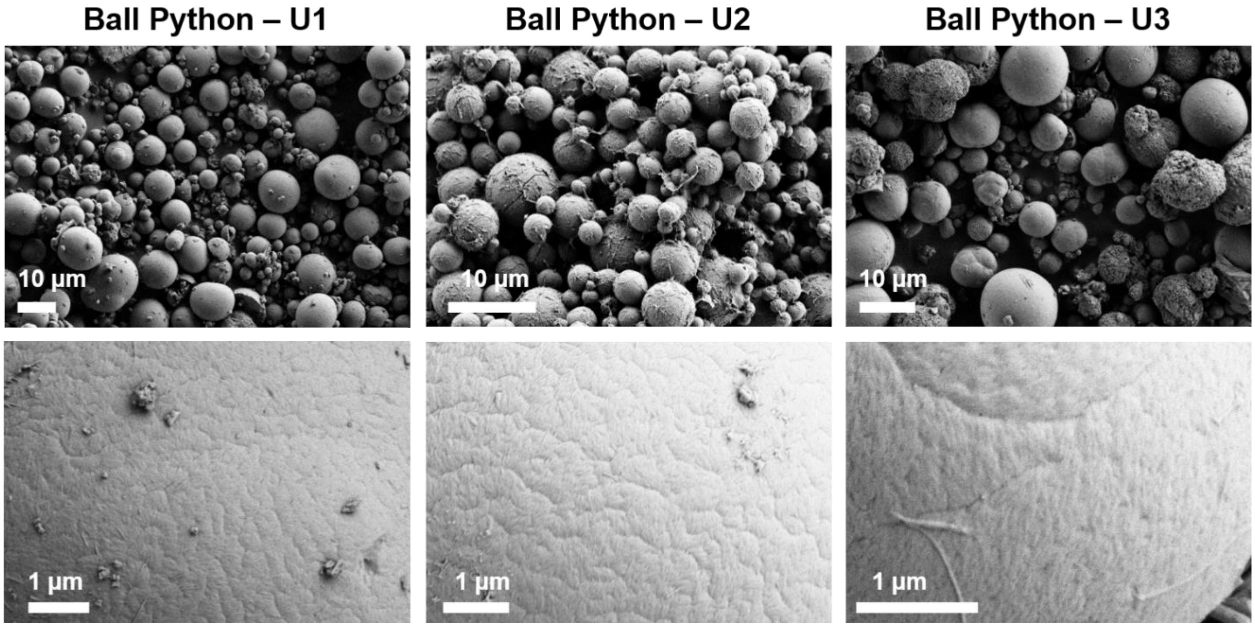

Figure S1. SEM images of Ball python urates U1, U2 and U3. There are no obvious visual differences in the sphere sizes or distribution in the U1 and U2 excretions. When U3 excretions are produced after a large meal, some of the microspheres (but not all) exhibit rougher surfaces. The roughening may be related to their partial transformation *in vivo*.

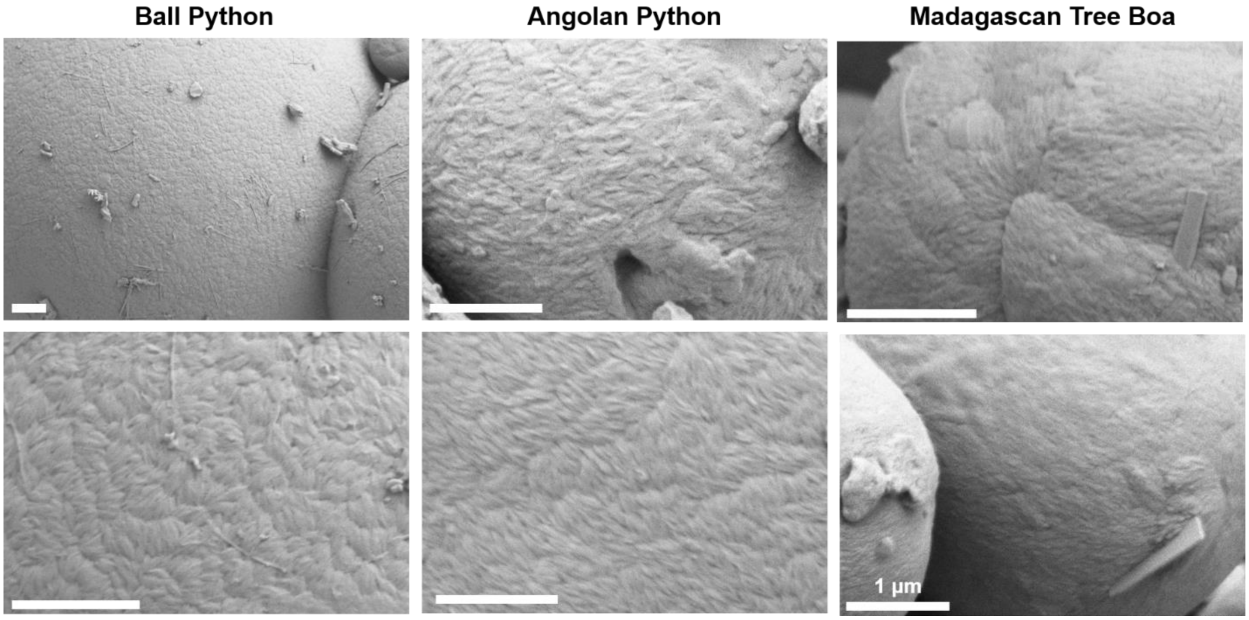

Ancient Snake Urates (sample age)	Average grain width (nm)		
Ball Python, U1 (21 days)	40	±	10
Ball Python, U2 (20 days)	40	±	20
Ball Python, U3 (13 days)	27	±	8
Ball Python, U1 (34 days)	50	±	10
Ball Python, U2 (30 days)	30	±	10
Ball Python, U3 (29 days)	40	±	10
Ball Python, U2 (3 years)	30	±	10
Angolan Python (3 years)	27	±	9
Madagascan Tree Boa (3 years)	30	±	10

Figure S2. (top) High-res SEM images of urates excreted by Ball python, Angolan python and Madagascan tree boa. All scale bars = 1 μm. (bottom) Average grain widths from multiple samples.

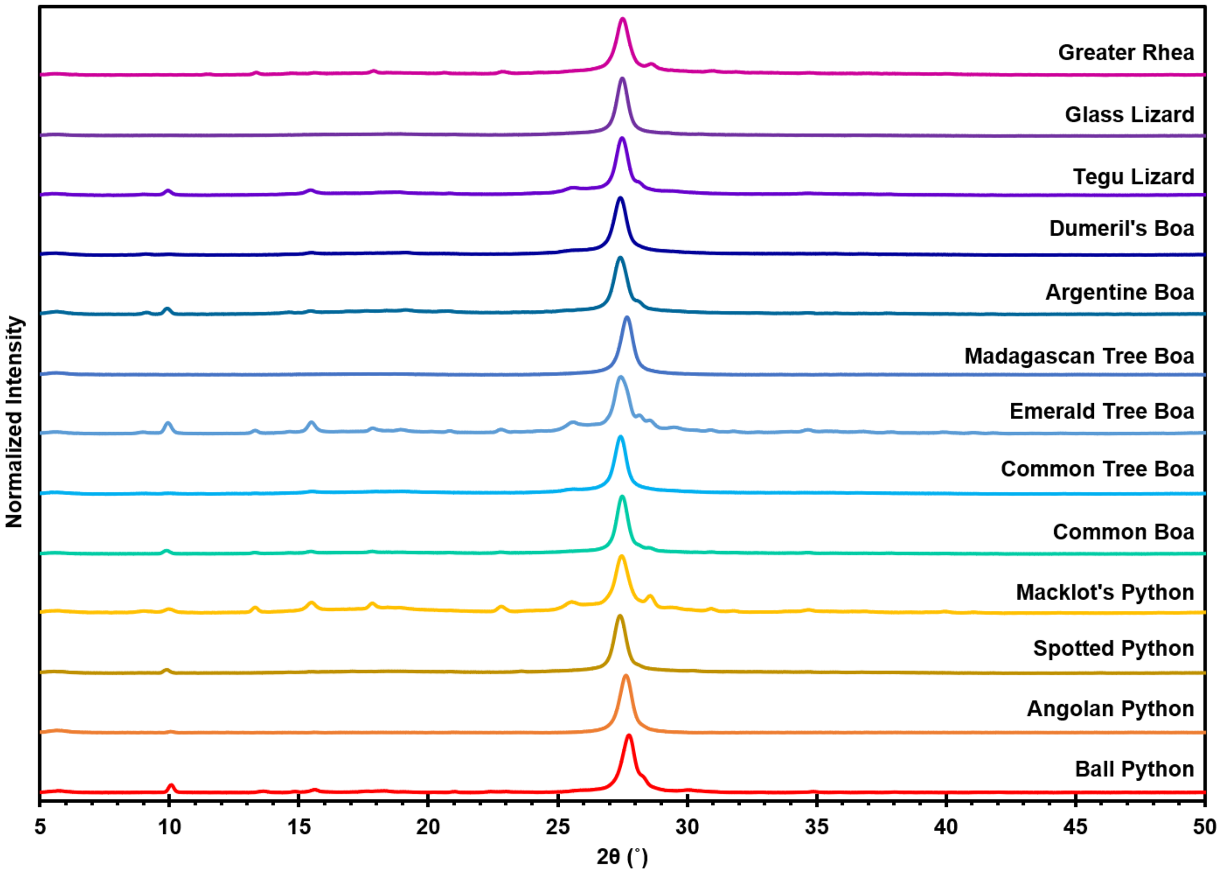

Figure S3. PXRD patterns of “urates” from 13 ancient avian and nonavian reptiles.

Table S2. Repeat distances between π-stacked units in uric acid and urate salt forms reported in the Cambridge Structural Database V5.43.

	refcode	π-stack repeat	Planar layer structure	2-theta (Miller index)
Uric acid (UA)	URICAC [71]	3.11 Å	N	28.84 (021)
Uric acid monohydrate (UAM)	GEJQAO [43]	3.20 Å	Y	27.84 (10-2)
Uric acid dihydrate (UAD)	ZZZPPI02 [25]	3.15 Å	N	28.35 (210)
Monosodium urate monohydrate (MSU)	NAURAT [76]	3.17 Å	Y	28.18 (011)
Lead urate	DITKEX [77]	3.04 Å	Y	
Calcium urate hexahydrate	YODJAE [78]	3.31 Å	Y	26.90 (020)
Magnesium urate (form I)	BADTEX10 [79]	3.25 Å	Y	
Magnesium urate (form II)	BADTEX01 [79]	3.36 Å	Y	
Potassium quadriurate	PABRIW [80]	3.60 Å	N	27.09 (11-3)<br>29.31 (11-4)
Ammonium urate (AU)	TIGZUI [67]	3.34 Å	Y	26.76 (11-2)
Ammonium urate hydrate (AUH)	RIVTEZ [60]	3.48 Å	Y	25.64 (10-1)
Methylene blue urate hexahydrate	UGEXIN [81]	3.25 Å	Y	
Guanidinium urate monohydrate	XANDEV [82]	3.39 Å	Y	
(2-aminopyridinium) urate dihydrate	MOYKAP [83]	3.45 Å	Y	
(2,6-diaminopyridinium) urate 1.5 hydrate	MOYGIT [83]	3.39 Å	N	
(2,4-diaminopyrimidinium) urate trihydrate	MOYKET [83]	3.57 Å	N	
(2,4,6-triaminopyrimidinium) urate tetrahydrate	MOYKIX [83]	3.61 Å	Y	

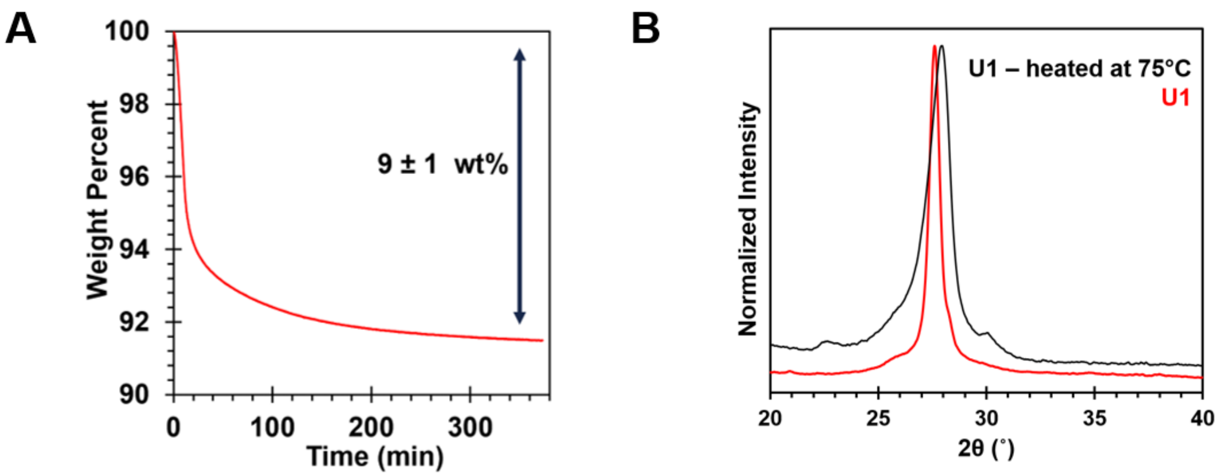

Figure S4. Isothermal heating of ball python urates under N_2 at 75°C shows (A) a ~9% weight by TGA. (B) The major peak in the PXRD pattern also becomes slightly asymmetric and shifts to a higher d-spacing = 3.225 Å after heating (calibrated against NIST SRM 640c).

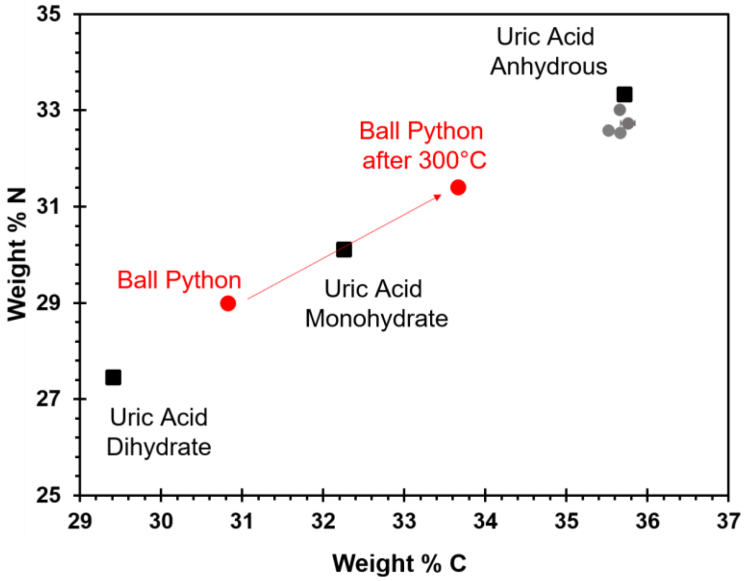

	C	H	N
**$C_5H_4N_4O_3$ (MW = 168)**	35.71%	2.38%	33.33%
**$C_5H_4N_4O_3 \cdot H_2O$ (MW = 186)**	32.26%	3.22%	30.10%
**$C_5H_4N_4O_3 \cdot 2\ H_2O$ (MW = 204)**	29.41%	3.92%	27.45%
**Expt. (before heating)**	30.83 ± 0.04 %	2.93 ± 0.01%	29.00 ± 0.01 %
**Expt. (heated 300°C)**	33.66 ± 0.02 %	2.29 ± 0.02 %	31.41 ± 0.04 %

Figure S5. Elemental analysis of ball python urates before and after heating at 10 °C /min to 300 °C (red circles) and uric acid (gray circles) compared against the calculated N/C ratios of uric acid anhydrate, monohydrate and dihydrate (black squares). Values are the average of three measurements. The initial sample is assumed to contain some non-crystalline water in addition to structural water.

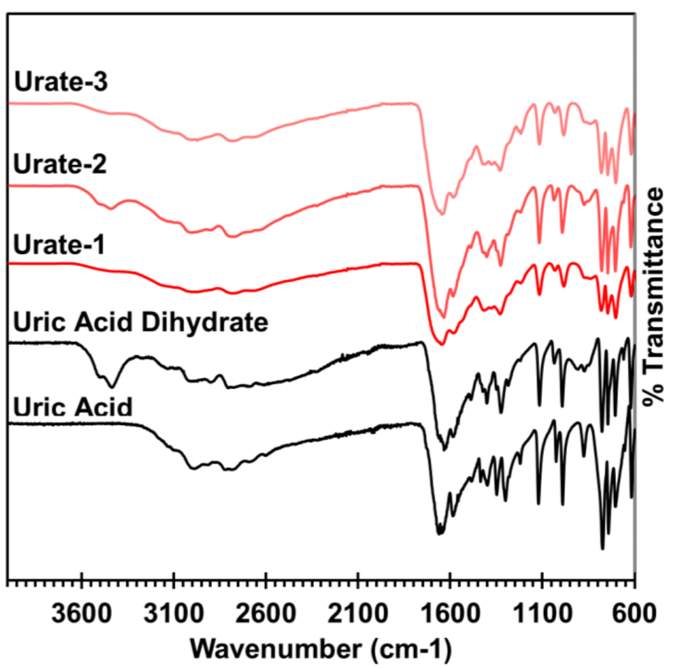

Figure S6. FT-IR spectra of ball python urates (U1, U2 and U3). The FT-IR spectrum of uric acid powder and uric acid dihydrate are included for reference. Uric acid dihydrate was grown according to the method reported in reference 81. Note: Sample U2 was confirmed by PXRD to contain some dihydrate.

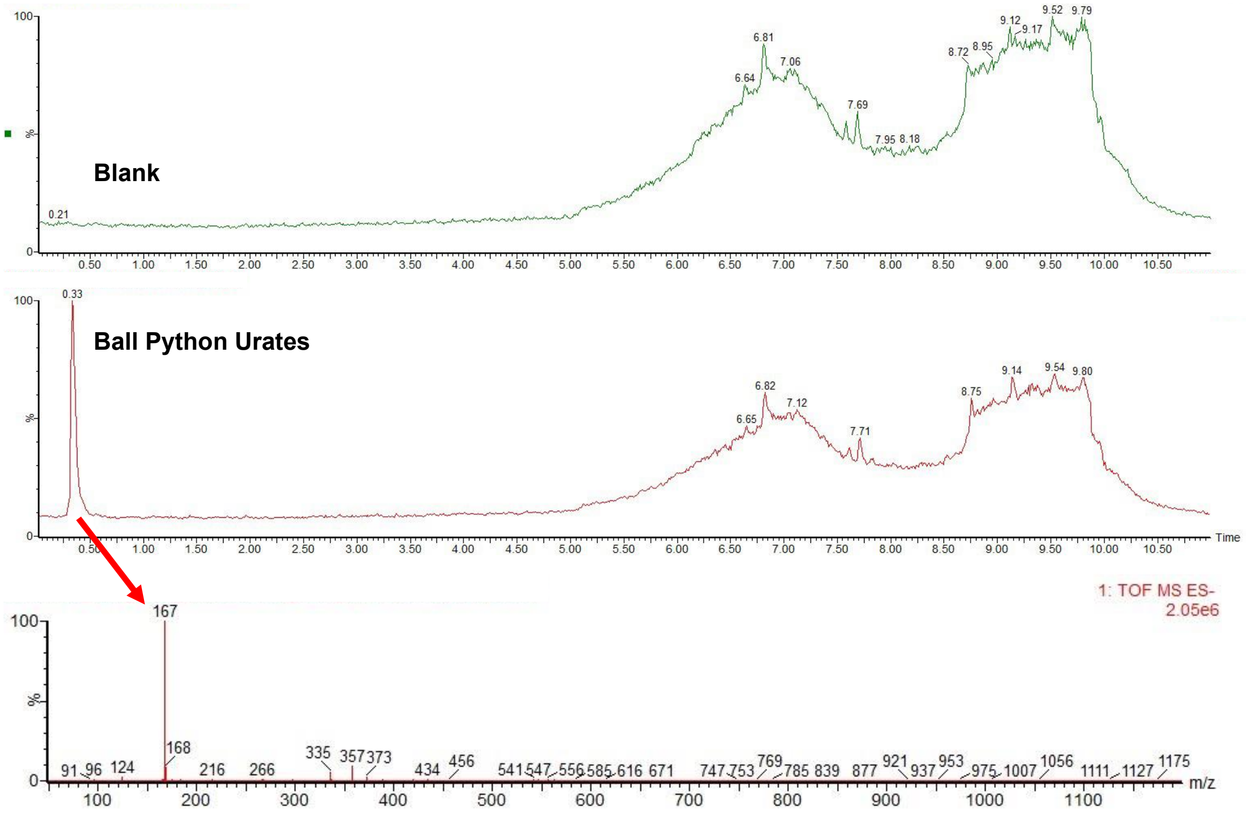

Figure S7. UPLC chromatograms and mass spectra of water blank and ball python urates sonicated in water in (-) ESI mode. Tandem MS-MS on the small peak at m/z = 357 indicated this was an adduct, $Na^+(urate^-)_2$.

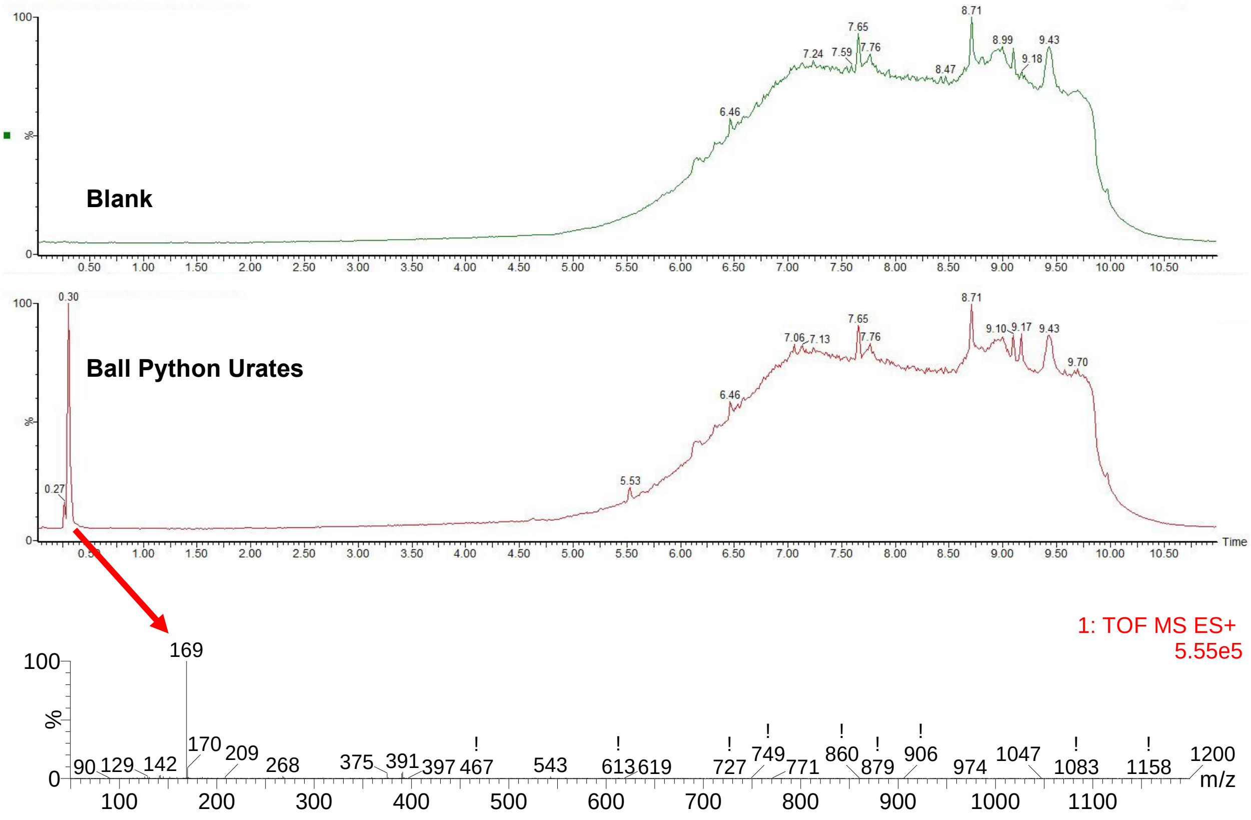

Figure S8. UPLC chromatograms and mass spectra of water blank and ball python urates sonicated in water in (+) ESI mode. The small peak at 5.53 minutes has a m/z ratio = 437, though its identity remains unknown.

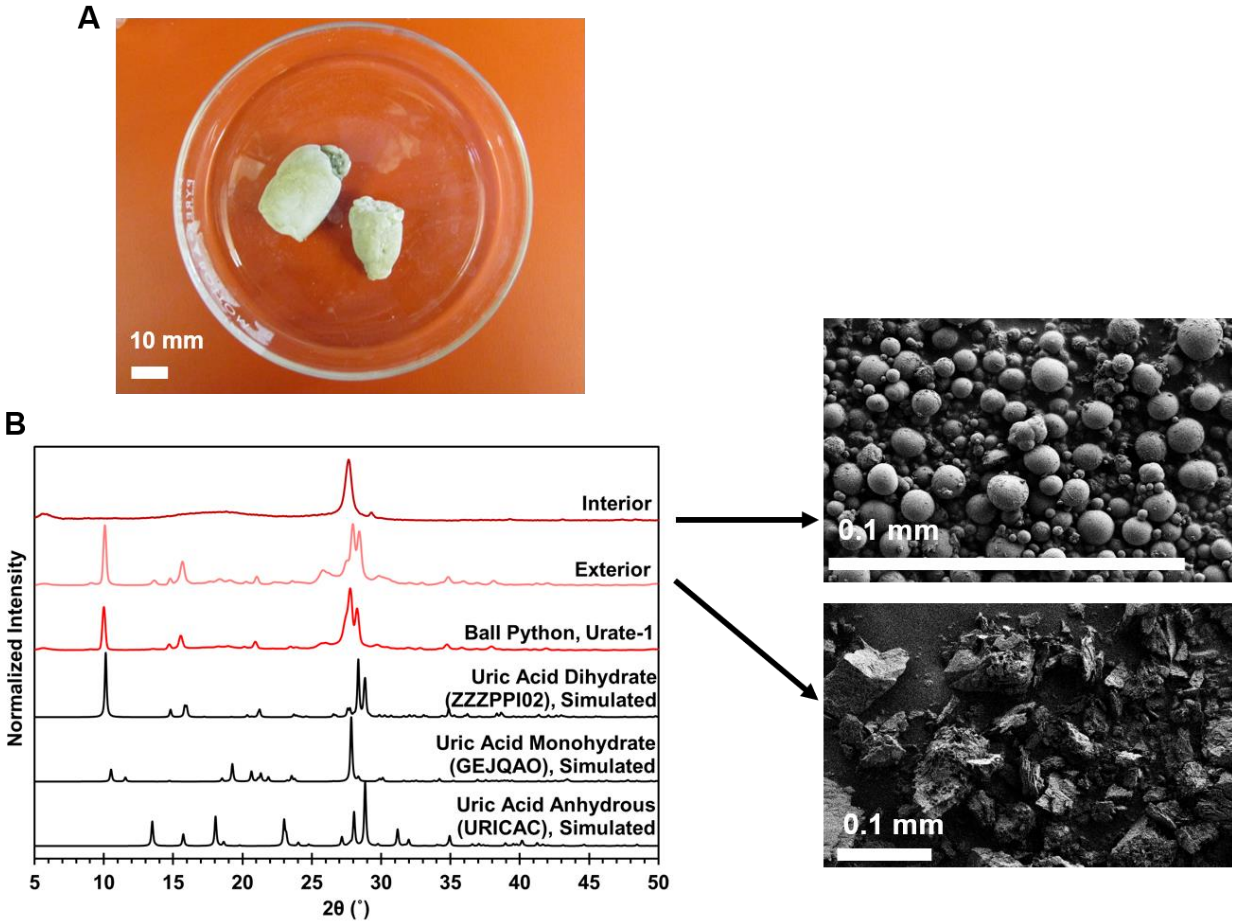

Figure S9. Under normal ambient storage conditions, some ball python urate samples show a slow partial transformation to UAD and UA. (A) From larger aged urate pellets, (B) fractions isolated from the interior retain the single-peak PXRD pattern and microsphere morphology, while material from the pellet exterior is faceted and has diffraction lines corresponding to more stable uric acid forms.

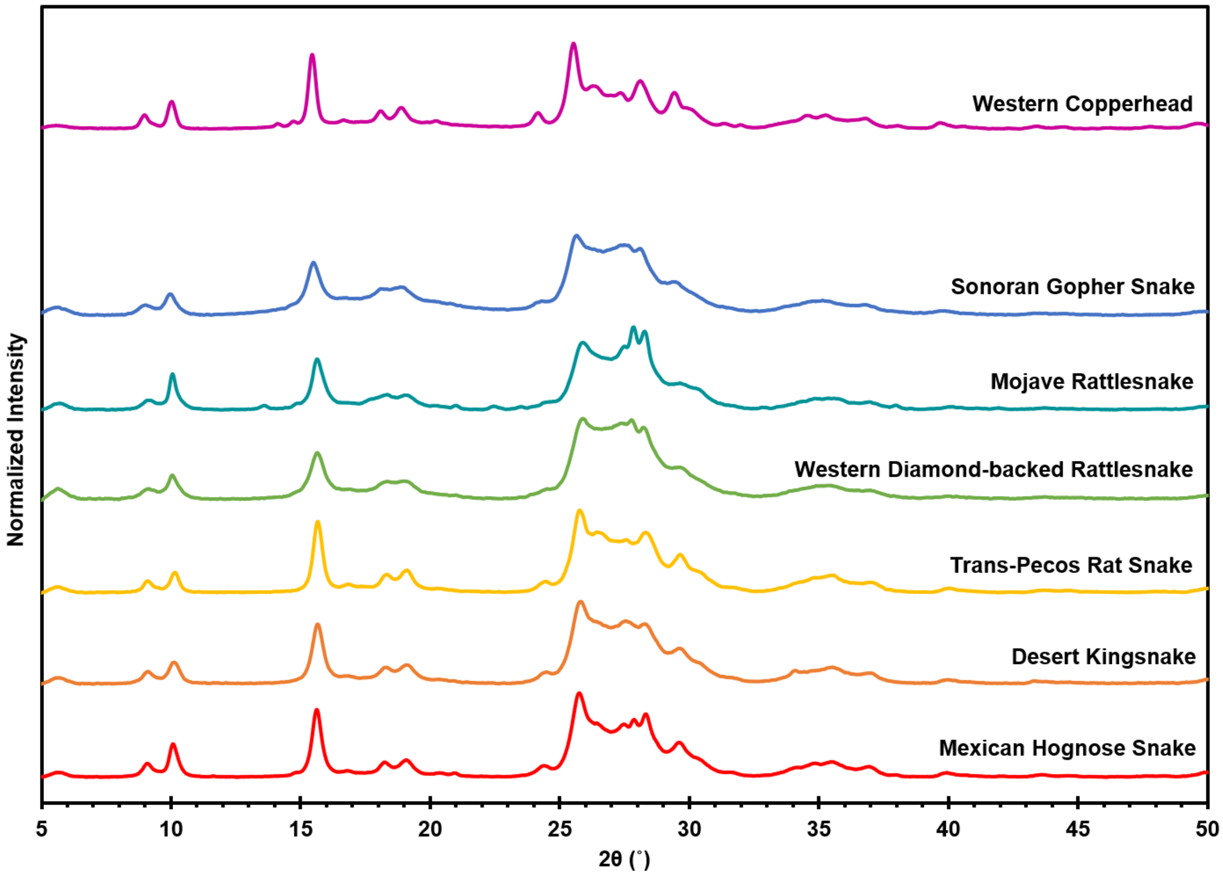

Figure S10. PXRD patterns of "urates" from snake species that excrete primarily ammonium urate include: Mexican hog-nosed snake (*Heterodon kennerlyi*), Desert kingsnake (*Lampropeltis splendida)*, Trans-Pecos ratsnake (*Bogertophis subocularis)*, Western diamond-backed rattlesnake (*Crotalus atrox)*, Mojave rattlesnake (*Crotalus scutulatus*), Sonoran gopher snake (*Pituophis catenifer*), and western copperhead (*Agkistrodon laticinctus)*. Mexican hog-nosed snake, western diamond-backed rattlesnake and Mojave rattlesnake contain small quantities of uric acid dihydrate evidenced by the two sharp diffraction lines at about 2-theta = 28°.

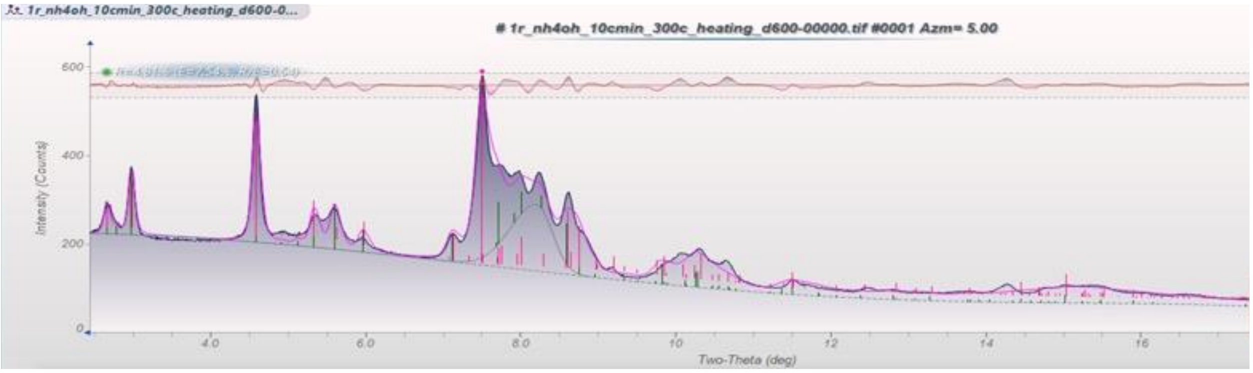

Figure S11. Rietveld refinement profiles of ball python spheres reacted in ammonium hydroxide (25% in water). Data was collected at the Advanced Photon Source beamline 17-BM-B and refined to an 86:14 mixture of ammonium urate hydrate:microspheres. The final R_{wp} = 4.79% (P-1: a = 3.661 Å, b = 10.106 Å, c = 10.598 Å, α = 113.91°, β = 90.6°, γ = 92.01°, V = 359.16 $Å^3$, Z = 2). The difference plot is shown at the top in red.

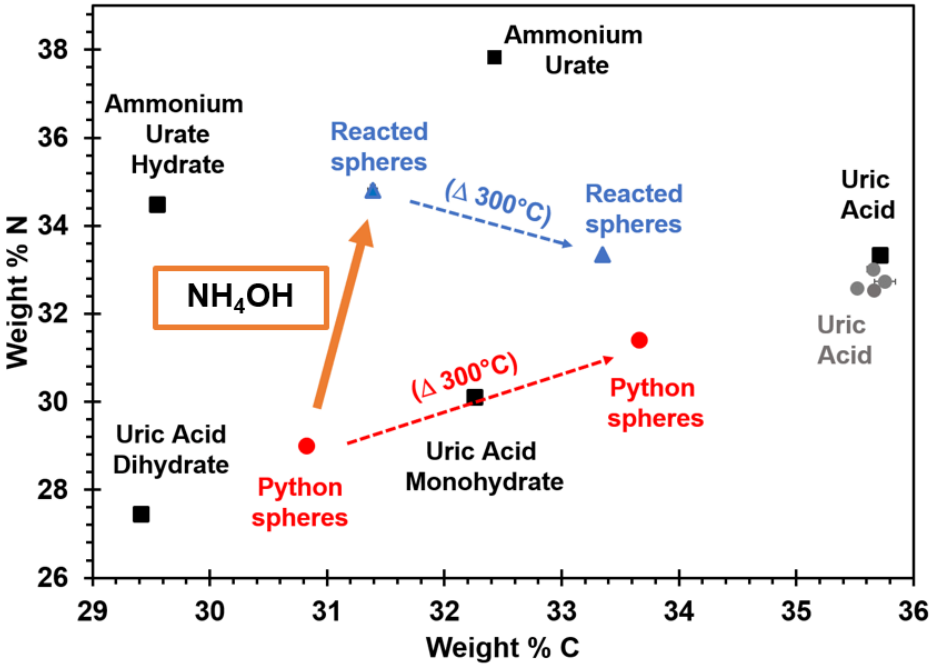

Figure S12. Elemental analysis of ball python microspheres before (red) and after (blue) immersion in an ammonium hydroxide solution and after heating at 10 °C /min to 300 °C. (black squares) Calculated N/C ratios for uric acid dihydrate, monohydrate and anhydrate, and ammonium urate monohydrate and anhydrate.

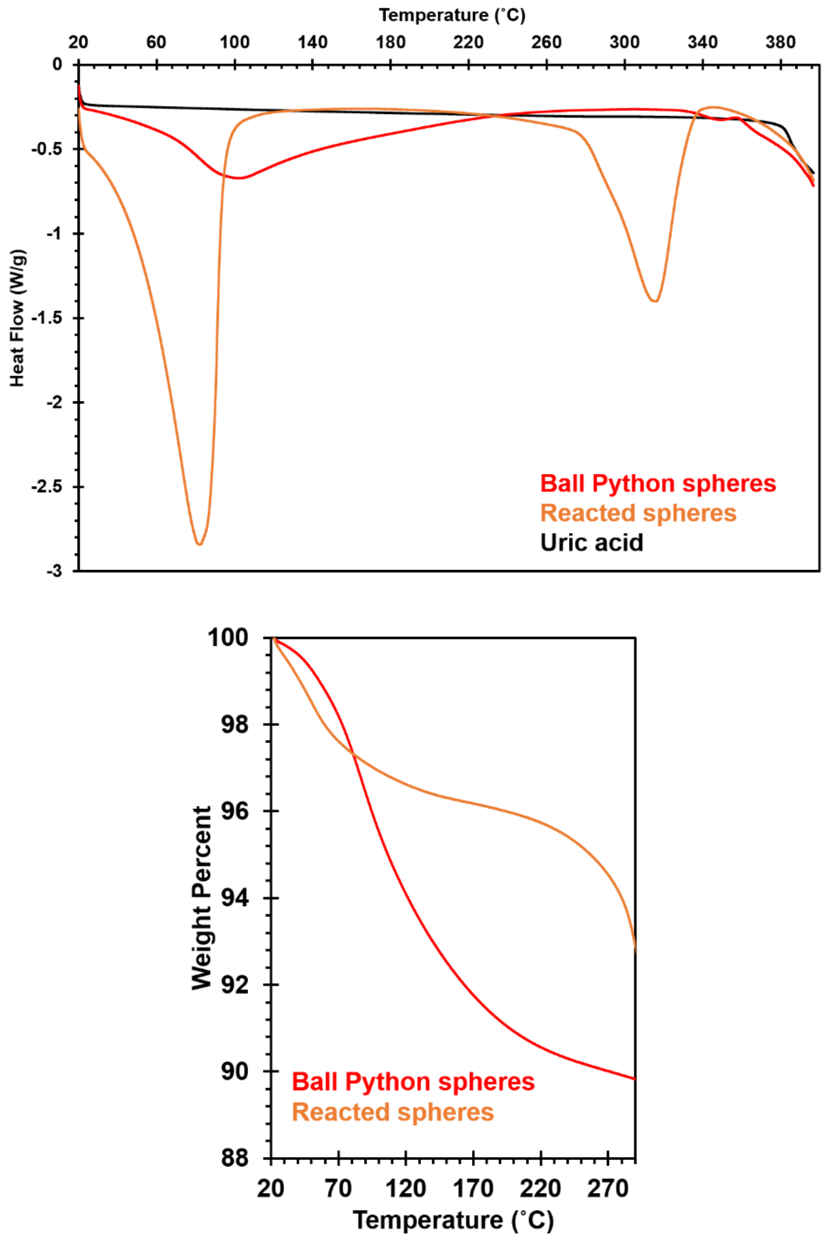

Figure S13. (top) DSC thermogram of ball python spheres, spheres after reaction in ammonium hydroxide solution, and uric acid. (bottom) TGA of the same ball python spheres and reacted spheres. Both samples were heated at 10 °C/min. For comparisons against synthetic material see reference 60.

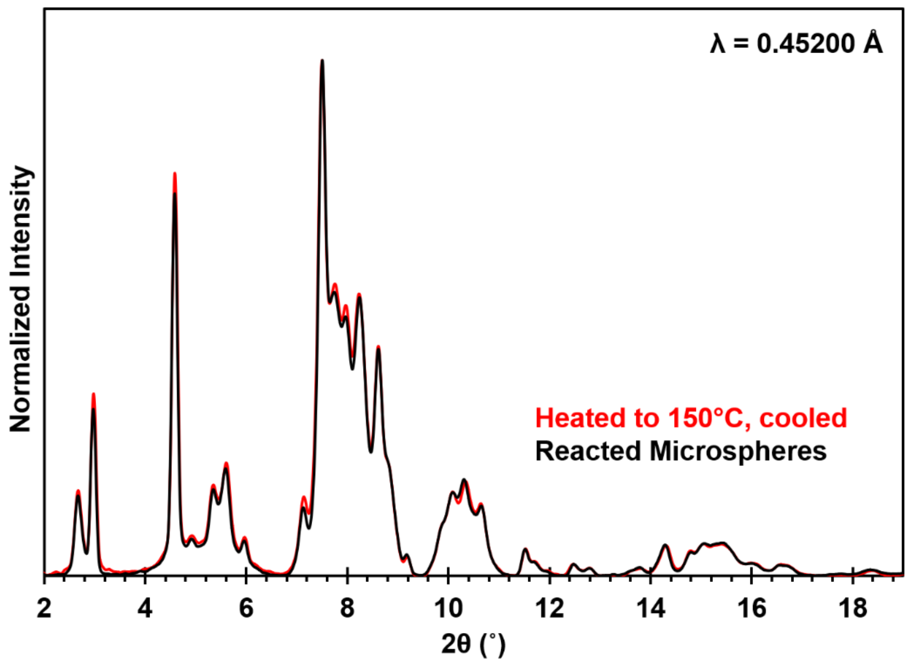

Figure S14. Overlay of room temperature synchrotron powder diffraction patterns of reacted ball python microspheres before (black) and after (red) heating to 150 °C. The absence of significant changes in the pattern indicates that the first endotherm in the DSC is due to loss of surface water not structural water. The 2θ scale corresponds to λ = 0.45200 Å.

References

1. Wilcox, W. R.; Khalaf, A.; Weinberger, A.; Kippen, I.; Klinenberg, J. R., Solubility of uric acid and monosodium urate. *Med. and Biol. Eng.* **1972,** *10*, 522-531.
2. Königsberger, E.; Wang, Z., Solubility of uric acid in salt solutions and artificial urine. *Monatshefte für Chemie* **1999,** *130*, 1067-73.
3. Fiske, C. H.; Boyden, E. A., Nitrogen metabolism in the chick embryo. *J. Biol. Chem.* **1926,** *70* (2), 535-556.
4. McNabb, R. A.; McNabb, F. M., Urate excretion by the avian kidney. *Comp. Biochem. Phys. A* **1975,** *51* (2), 253-8.
5. Bardin, T.; Richette, P., Definition of hyperuricemia and gouty conditions. *Curr. Opin. Rheumatol.* **2014,** *26* (2), 186-191.
6. Mattiuzzi, C.; Lippi, G., Recent updates on worldwide gout epidemiology. *Clin. Rheumatol.* **2020,** *39* (4), 1061-1063.
7. Danve, A.; Neogi, T., Rising Global Burden of Gout: Time to Act. *Arthritis Rheumatol.* **2020,** *72* (11), 1786-1788.
8. Dehlin, M.; Jacobsson, L.; Roddy, E., Global epidemiology of gout: prevalence, incidence, treatment patterns and risk factors. *Nat. Rev. Rheumatol.* **2020,** *16* (7), 380-390.
9. Thongprayoon, C.; Krambeck, A. E.; Rule, A. D., Determining the true burden of kidney stone disease. *Nat. Rev. Nephrol.* **2020,** *16* (12), 736-746.

10. Oda, M.; Satta, Y.; Takenaka, O.; Takahata, N., Loss of urate oxidase activity in hominoids and its evolutionary implications *Mol. Biol. Evol.* **2002,** *19*, 640-653.
11. Kratzer, J. T.; Lanaspa, M. A.; Murphy, M. N.; Cicerchi, C.; Graves, C. L.; Tipton, P. A.; Ortlund, E. A.; Johnson, R. J.; Gaucher, E. A., Evolutionary history and metabolic insights of ancient mammalian uricases. *Proc. Natl. Acad. Sci. U.S.A.* **2014,** *111* (10), 3763-3768.
12. Wu, X.; Muzny, D. M.; Chi Lee, C.; Caskey, C. T., Two independent mutational events in the loss of urate oxidase during hominoid evolution. *J. Mol. Evol.* **1992,** *34* (1), 78-84.
13. Johnson, R. J.; Stenvinkel, P.; Andrews, P.; Sánchez-Lozada, L. G.; Nakagawa, T.; Gaucher, E.; Andres-Hernando, A.; Rodriguez-Iturbe, B.; Jimenez, C. R.; Garcia, G.; Kang, D.-H.; Tolan, D. R.; Lanaspa, M. A., Fructose metabolism as a common evolutionary pathway of survival associated with climate change, food shortage and droughts. *J. Intern. Med.* **2020,** *287* (3), 252-262.
14. Li, Z.; Hoshino, Y.; Tran, L.; Gaucher, E. A., Phylogenetic Articulation of Uric Acid Evolution in Mammals and How It Informs a Therapeutic Uricase. *Mol. Bio. Evol.* **2021,** *39* (1).
15. Moe, O. W., Uric acid nephrolithiasis: Proton titration of an essential molecule? *Curr. Opin. Nephrol. Hy.* **2006,** *15*, 366-373.
16. Ames, B. N.; Cathcart, R.; Schwiers, E.; Hochstein, P., Uric acid provides an antioxidant defense in humans against oxidant- and radical-caused aging and cancer: a hypothesis. *Proc. Natl. Acad. Sci. U.S.A.* **1981,** *78* (11), 6858-62.
17. Shi, Y.; Evans, J. E.; Rock, K. L., Molecular identification of a danger signal that alerts the immune system to dying cells. *Nature* **2003,** *425* (6957), 516-521.
18. Ord, W. M., *On the Influence of Colloids upon Crystalline Form and Cohesion*. Edward Stanford: London, 1879.
19. Roberts, W., On the History of Uric Acid in the Urine, with reference to the Formation of Uric Acid Concretions and Deposits. *Medico-chirurgical transactions* **1890,** *73*, 245-71.
20. Folk, R. L., Spherical Urine in Birds: Petrography. *Science* **1969,** *166* (3912), 1516-1518.
21. Poulson, T. L.; McNabb, F. M. A., Uric Acid: The Main Nitrogenous Excretory Product of Birds. *Science* **1970,** *170*, 98-99.
22. Willoughby, E. J., Composition of avian urine. *Science* **1970,** *169* (3951), 1230-1231.
23. Lonsdale, K.; Sutor, D. J., Uric acid dihydrate in bird urine. *Science* **1971,** *172*, 958-959.
24. Artioli, G.; Masciocchi, N.; Galli, E., The elusive crystal structure of uric acid dihydrate: implication for epitaxial growth during biomineralization. *Acta Crystallogr. B* **1997,** *53*, 498-503.
25. Parkin, S.; Hope, H., Uric acid dihydrate revisited. *Acta. Crystallogr. B* **1998,** *54*, 339-344.
26. Boykin, S. Relationship between Protein and Urate in Avian Urine. University of Arizona PhD Thesis, 1995.
27. Drees, L. R.; Manu, A., Bird urate contamination of atmospheric dust traps. *CATENA* **1996,** *27* (3–4), 287-294.
28. Casotti, G.; Braun, E. J., Protein location and elemental composition of urine spheres in different avian species. *J. Exp. Zool. Part A* **2004,** *301A* (7), 579-587.
29. Crouch, N. M. A.; Lynch, V. M.; Clarke, J. A., A re-evaluation of the chemical composition of avian urinary excreta. *J. Ornithol.* **2020,** *161* (1), 17-24.
30. Minnich, J. E.; Piehl, P. A., Spherical precipitates in the urine of reptiles. *Comp. Biochem. Phys. A* **1972,** *41* (3), 551-554.
31. Goh, K.-S.; Sheu, H.-S.; Hua, T.-E.; Kang, M.-H.; Li, C.-L., Uric Acid Spherulites in the Reflector Layer of Firefly Light Organ. *PloS One* **2013,** *8* (2), e56406.
32. Thornton, A. M.; Schuett, G. W.; Swift, J. A., Urates of colubroid snakes are different from those of boids and pythonids. *Biol. J. Linn. Soc.* **2021,** *133* (3), 910-919.
33. Tojo, S.; Betchaku, T.; Ziccardi, V. J.; Wyatt, G. R., Fat body protein granules and storage proteins in the silkmoth, Hyalophora cecropia. *J. Cell Biol.* **1978,** *78* (3), 823-838.
34. Mullins, D. E., Isolation and partial characterization of uric acid spherules obtained from cockroach tissues (Dictyoptera). *Comp. Biochem. Phys. A* **1979,** *62* (3), 699-705.

35. Ninomiya Y, T. K., Hayakawa Y., Mechanisms of black and white stripe pattern formation in the cuticles of insect larvae. *J. Insect Physiol.* **2006,** *52* (6), 638-645.
36. Burbrink, F. T.; Grazziotin, F. G.; Pyron, R. A.; Cundall, D.; Donnellan, S.; Irish, F.; Keogh, J. S.; Kraus, F.; Murphy, R. W.; Noonan, B.; Raxworthy, C. J.; Ruane, S.; Lemmon, A. R.; Lemmon, E. M.; Zaher, H., Interrogating Genomic-Scale Data for Squamata (Lizards, Snakes, and Amphisbaenians) Shows no Support for Key Traditional Morphological Relationships. *Systematic Biol.* **2020,** *69* (3), 502-520.
37. Porter, P., Physico-chemical Factors Involved in Urate Calculus Formation: II.—Colloidal Flocculation. *Res. Vet. Sci.* **1963,** *4* (4), 592-602.
38. Porter, P., Colloidal Properties of Urates in Relation to Calculus Formation. *Res. Vet. Sci.* **1966,** *7* (2), 128-138.
39. McNabb, R. A.; McNabb, F. M. A., Physiological chemistry of uric acid: Solubility, colloid, and ion-binding properties. *Comp. Biochem. Physiol. A* **1980,** *67*, 27-34.
40. Bates, S.; Zografi, G.; Engers, D.; Morris, K.; Crowley, K.; Newman, A., Analysis of Amorphous and Nanocrystalline Solids from Their X-Ray Diffraction Patterns. *Pharm. Res.* **2006,** *23* (10), 2333-2349.
41. Li, Z. Q.; Lu, C. J.; Xia, Z. P.; Zhou, Y.; Luo, Z., X-ray diffraction patterns of graphite and turbostratic carbon. *Carbon* **2007,** *45* (8), 1686-1695.
42. Viani, A.; Gualtieri, A. F.; Artioli, G., The nature of disorder in montmorillonite by simulation of X-ray powder patterns. *Am. Mineral.* **2002,** *87* (7), 966-975.
43. Schubert, G.; Reck, G.; Jancke, H.; Kraus, W.; Patzelt, C., Uric acid monohydrate - a new urinary calculus phase. *Urol. Res.* **2005,** *33* (3), 231-238.
44. Pinsk, N.; Wagner, A.; Cohen, L.; Smalley, C. J. H.; Hughes, C. E.; Zhang, G.; Pavan, M. J.; Casati, N.; Jantschke, A.; Goobes, G.; Harris, K. D. M.; Palmer, B. A., Biogenic Guanine Crystals Are Solid Solutions of Guanine and Other Purine Metabolites. *J. Am. Chem. Soc.* **2022,** *144* (11), 5180-5189.
45. Henze, M. J.; Lind, O.; Wilts, B. D.; Kelber, A., Pterin-pigmented nanospheres create the colours of the polymorphic damselfly *Ischnura elegans*. *J. R. Soc. Interface.* **2019,** *16* (153), 20180785.
46. Wilts, B. D.; Wijnen, B.; Leertouwer, H. L.; Steiner, U.; Stavenga, D. G., Extreme Refractive Index Wing Scale Beads Containing Dense Pterin Pigments Cause the Bright Colors of Pierid Butterflies. *Advanced Optical Materials* **2017,** *5* (3), 1600879.
47. Palmer, B. A.; Yallapragada, V. J.; Schiffmann, N.; Wormser, E. M.; Elad, N.; Aflalo, E. D.; Sagi, A.; Weiner, S.; Addadi, L.; Oron, D., A highly reflective biogenic photonic material from core–shell birefringent nanoparticles. *Nature Nanotechnology* **2020,** *15* (2), 138-144.
48. Lemcoff, T.; Alus, L.; Haataja, J. S.; Wagner, A.; Zhang, G.; Pavan, M. J.; Yallapragada, V. J.; Vignolini, S.; Oron, D.; Schertel, L.; Palmer, B. A., Brilliant whiteness in shrimp from ultra-thin layers of birefringent nanospheres. *Nature Photonics* **2023**.
49. Barzilay, Y.; Eyal, Z.; Noy, Y.; Varsano, N.; Olender, T.; Bera, S.; Lerer-Goldshtein, T.; Kedmi, M.; Porat, Z.; Pinkas, I.; Levin-Zaidman, S.; Dezorella, N.; Gur, D., Specialized molecular pathways drive the formation of light-scattering assemblies in leucophores. *Proc. Natl. Acad. Sci. U.S.A.* **2025,** *122* (22), e2424979122.
50. V Shoemaker, I., and; Nagy, K. A., Osmoregulation in Amphibians and Reptiles. *Annual Review of Physiology* **1977,** *39* (1), 449-471.
51. Dantzler, W. H., Renal Mechanisms for Osmoregulation in Reptiles and Birds. In *Animals and Environmental Fitness: Physiological and Biochemical Aspects of Adaptation and Ecology*, Gilles, R., Ed. Pergamon: 1980; pp 91-110.
52. Casotti, G.; Braun, E. J., Ionic composition of urate-containing spheres in the urine of domestic fowl. *Comp. Biochem. Phys. A* **1997,** *118* (3), 585-588.
53. Bradley, T. J., *Animal Osmoregulation*. Oxford 2008.
54. Presores, J. B.; Swift, J. A., Adhesion Properties of Uric Acid Crystal Surfaces. *Langmuir* **2012,** *28* (19), 7401-7406.

55. Presores, J. B.; Swift, J. A., Solution-mediated phase transformation of uric acid dihydrate. *CrystEngComm* **2014,** *16* (31), 7278-7284.
56. https://reptile-database.reptarium.cz/, The Reptile Database.
57. Bartges, J.; Osborne, C. A.; Lulich, J. P.; Unger, L.; Koehler, L.; Bird, K.; Clinton, C.; Davenport, M., Prevalence of cystine and urate uroliths in bulldogs and urate uroliths in dalmatians. *J. Am. Vet. Med. Assoc.* **1994,** *204* (12), 1914-1918.
58. Friedel, P.; Bergmann, J.; Kleeberg, R.; Schubert, G., A proposition for the structure of ammonium hydrogen (acid) urate from uroliths. *Z. Kristallogr. Suppl.* **2006,** *23*, 517-22.
59. Geng, X.; Meegan, J.; Smith, C.; Sakhaee, K.; Rimer, J. D., Crystallization of Hierarchical Ammonium Urate: Insight into the Formation of Cetacean Renal Stones. *Cryst. Growth Des.* **2019,** *19* (11), 6727-6735.
60. Thornton, A. M.; Fawcett, T. G.; Kaduk, J. A.; Lin, Y.; Swift, J. A., An Improved Model for Biogenic Ammonium Urate *Cryst. Growth Des.* **2023,** *23*, 6953-6959.
61. Griffin, D. K.; Larkin, D. M.; O'Connor, R. E.; Romanov, M. N., Dinosaurs: Comparative Cytogenomics of Their Reptile Cousins and Avian Descendants. *Animals* **2023,** *13* (1), 106.
62. Organ, C. L.; Shedlock, A. M.; Meade, A.; Pagel, M.; Edwards, S. V., Origin of avian genome size and structure in non-avian dinosaurs. *Nature* **2007,** *446* (7132), 180-184.
63. Braun, E. J.; Campbell, C. E., Uric acid decomposition in the lower gastrointestinal tract. *J. Exp. Zool.* **1989,** *252* (S3), 70-74.
64. Wang, Z.; Königsberger, L.; Königsberger, E., Solubility equilibria in the uric acid–ammonium urate–water system. *Monatshefte für Chemie* **2018,** *149* (2), 327-332.
65. Crawley, W. T.; Jungels, C. G.; Stenmark, K. R.; Fini, M. A., U-shaped association of uric acid to overall-cause mortality and its impact on clinical management of hyperuricemia. *Redox Biol.* **2022,** *51*, 102271.
66. Allegrini, S.; Garcia-Gil, M.; Pesi, R.; Camici, M.; Tozzi, M. G., The Good, the Bad and the New about Uric Acid in Cancer. *Cancers* **2022,** *14* (19), 4959.
67. Tang, W.; Yang, T.; Morales-Rivera, C. A.; Geng, X.; Srirambhatla, V. K.; Kang, X.; Chauhan, V. P.; Hong, S.; Tu, Q.; Florence, A. J.; Mo, H.; Calderon, H. A.; Kisielowski, C.; Hernandez, F. C. R.; Zou, X.; Mpourmpakis, G.; Rimer, J. D., Tautomerism unveils a self-inhibition mechanism of crystallization. *Nat. Commun.* **2023,** *14* (1), 561.
68. Perrin, C. M.; Dobish, M. A.; Van Keuren, E.; Swift, J. A., Monosodium urate monohydrate crystallization. *CrystEngComm* **2011,** *13* (4), 1111-1117.
69. Chupas, P. J.; Chapman, K. W.; Kurtz, C.; Hanson, J. C.; Lee, P. L.; Grey, C. P., A versatile sample-environment cell for nonambient X-ray scattering experiments. *J. Appl. Crystallogr.* **2008,** *41*, 822-824.
70. Toby, B. H.; Von Dreele, R. B., GSAS-II: the genesis of a modern open-source all purpose crystallography software package. *J. Appl. Crystallogr.* **2013,** *46* (2), 544-549.
71. Ringertz, H., The molecular and crystal structure of uric acid. *Acta Crystallographica* **1966,** *20*, 397-403.
72. Taylor, R.; Wood, P. A., A Million Crystal Structures: The Whole Is Greater than the Sum of Its Parts. *Chem. Rev.* **2019,** *119*, 9427-9477.
73. Fawcett, T. G.; Kabekkodu, S. N.; Blanton, J. R.; Blanton, T. N., Chemical analysis by diffraction: the Powder Diffraction File. *Powder Diffr.* **2017,** *32* (2), 63-71.
74. Gates-Rector, S.; Blanton, T., The Powder Diffraction File: a quality materials characterization database. *Powder Diffr.* **2019,** *34* (4), 352-360.
75. Kresse, G.; Furthmüller, J., Efficiency of ab-initio total energy calculations for metals and semiconductors using a plane-wave basis set. *Comp. Mater. Sci.* **1996,** *6* (1), 15-50.
76. Mandel, N. S.; Mandel, G. S., Monosodium urate monohydrate, the gout culprit. *J. Am. Chem. Soc.* **1976,** *98* (8), 2319-2323.

77. Sattar, S.; Carroll, M. J.; Sargeant, A. A.; Swift, J. A., Structure of a lead urate complex and its effect on the nucleation of monosodium urate monohydrate. *CrystEngComm* **2008,** *10* (2), 155-157.
78. Presores, J. B.; Cromer, K. E.; Capacci-Daniel, C.; Swift, J. A., Calcium Urate Hexahydrate. *Cryst. Growth Des.* **2013,** *13* (12), 5162-5164.
79. Dubler, E.; Jameson, G. B.; Kopajtic, Z., Uric acid salts of magnesium: Crystals and molecular structures and thermal analysis of two phases of $Mg(C_5H_3N_4O_3)_2 \cdot 8H_2O$. *J. Inorg. Biochem.* **1986,** *26*, 1-21.
80. Bazin, D.; Daudon, M.; Elkaim, E.; Le Bail, A.; Smrčok, Ĺ., Ab initio structure determination of kidney stone potassium quadriurate from synchrotron powder diffraction data, a 150 year problem solved. *Comptes Rendus Chimie* **2016,** *19* (11-12), 1535-1541.
81. Sours, R. E.; Fink, D. A.; Swift, J. A., Dyeing uric acid crystals with methylene blue. *J. Am. Chem. Soc.* **2002,** *124*, 8630-8636.
82. Poje, N.; Poje, M.; Vickovic, I., Crystal structure of guanidinium urate monohydrate, C5H3N4O3(CH6N3) · H2O. *Z. Kristallogr. NCS* **2000,** *215*, 583-584.
83. Hall, V. M.; Thornton, A.; Miehls, E. K.; Bertke, J. A.; Swift, J. A., Uric Acid Crystallization Interrupted with Competing Binding Agents. *Cryst. Growth Des.* **2019,** *19* (12), 7363-7371.
